# The lysophosphatidic acid-regulated signal transduction network in ovarian cancer cells and its role in actomyosin dynamics, cell migration and entosis

**DOI:** 10.7150/thno.81656

**Published:** 2023-03-21

**Authors:** Kaire Ojasalu, Sonja Lieber, Anna M. Sokol, Andrea Nist, Thorsten Stiewe, Imke Bullwinkel, Florian Finkernagel, Silke Reinartz, Sabine Müller-Brüsselbach, Robert Grosse, Johannes Graumann, Rolf Müller

**Affiliations:** 1 Department of Translational Oncology, Center for Tumor Biology and Immunology, Philipps University, Marburg, Germany; 2 Biomolecular Mass Spectrometry, Max-Planck-Institute for Heart and Lung Research, Bad Nauheim, Germany; 3 Genomics Core Facility, Philipps University, Marburg, Germany; 4 Bioinformatics Core Facility, Philipps University, Marburg, Germany; 5 Institut for Experimental and Clinical Pharmacology and Toxicology, Albert-Ludwigs University, Freiburg, Germany; 6 Institute for Translational Proteomics, Philipps University, Marburg, Germany

**Keywords:** entosis, migration, lysophosphatidic acid, DOCK7, MYPT1

## Abstract

Lysophosphatidic acid (LPA) species accumulate in the ascites of ovarian high-grade serous cancer (HGSC) and are associated with short relapse-free survival. LPA is known to support metastatic spread of cancer cells by activating a multitude of signaling pathways via G-protein-coupled receptors of the LPAR family. Systematic unbiased analyses of the LPA-regulated signal transduction network in ovarian cancer cells have, however, not been reported to date.

**Methods:** LPA-induced signaling pathways were identified by phosphoproteomics of both patient-derived and OVCAR8 cells, RNA sequencing, measurements of intracellular Ca^2+^ and cAMP as well as cell imaging. The function of LPARs and downstream signaling components in migration and entosis were analyzed by selective pharmacological inhibitors and RNA interference.

**Results:** Phosphoproteomic analyses identified > 1100 LPA-regulated sites in > 800 proteins and revealed interconnected LPAR1, ROCK/RAC, PKC/D and ERK pathways to play a prominent role within a comprehensive signaling network. These pathways regulate essential processes, including transcriptional responses, actomyosin dynamics, cell migration and entosis. A critical component of this signaling network is MYPT1, a stimulatory subunit of protein phosphatase 1 (PP1), which in turn is a negative regulator of myosin light chain 2 (MLC2). LPA induces phosphorylation of MYPT1 through ROCK (T853) and PKC/ERK (S507), which is majorly driven by LPAR1. Inhibition of MYPT1, PKC or ERK impedes both LPA-induced cell migration and entosis, while interference with ROCK activity and MLC2 phosphorylation selectively blocks entosis, suggesting that MYPT1 figures in both ROCK/MLC2-dependent and -independent pathways. We finally show a novel pathway governed by LPAR2 and the RAC-GEF DOCK7 to be indispensable for the induction of entosis.

**Conclusion:** We have identified a comprehensive LPA-induced signal transduction network controlling LPA-triggered cytoskeletal changes, cell migration and entosis in HGSC cells. Due to its pivotal role in this network, MYPT1 may represent a promising target for interfering with specific functions of PP1 essential for HGSC progression.

## Introduction

Ovarian cancer (OC) ranks fifth in cancer deaths among women and represents the most lethal cancer of the female reproductive system 1, 2. Its most aggressive form is high-grade serous carcinoma (HGSC), which accounts for the majority of ovarian malignancies. Due to a lack of specific symptoms, HGSC is frequently diagnosed at advanced stages with widespread peritoneal metastases, resulting in a dire 5-year survival rate of less than 30% 2, 3. A characteristic feature of HGSC is its tumor microenvironment 4, 5, which is composed of solid tumor masses in peritoneal organs and the peritoneal fluid (ascites at advanced stages). Tumor cell spheroids in the latter play a causative role in metastatic spread 4, 5.

Besides tumor cells, ascites abounds with immune cells, which produce a plethora of soluble mediators with metastasis-promoting and immune suppressive properties 4-7. Among these is lysophosphatidic acid (LPA), originally termed “ovarian cancer activating factor” 8. LPA represents a group of pro-tumorigenic lipids 9, 10 consisting of a glycerol backbone with a fatty acid in the sn1 or sn2 position and a phosphate group in sn3 as well as variable length and saturation of the fatty acid chains 11. It is noteworthy that the majority of LPA research has been carried out with 18:1-LPA, although ascites is dominated by 20:4 and 18:2 acyl-LPAs followed by 16:0, 18:1 and 18:0 species 12. In HGSC, extracellular LPA is generated from phospholipids by the consecutive action of secretory phospholipase A_2_ (in particular PLA2G7) and the lysophospholipase D autotaxin 9, 13. Its degradation is mediated by lipid phosphate phosphatases, known to be downregulated in the majority of ovarian cancers 14.

LPA signals through at least six G-protein-coupled receptors (GPCRs) termed LPAR1-6, which trigger both overlapping and distinct signaling pathways, including phospholipase C, phosphatidylinositol 3-kinase, adenylate cyclase and Rac/Rho, by involving at least two, if not all four Gα proteins 15. LPARs can be divided into two families. LPAR1-3 are classified as members of the endothelial cell differentiation gene (EDG) subfamily of GPCRs, while the other three LPA receptors are structurally more closely related to the purinergic receptors, yet do not bind nucleotides 15. LPAR1 is the most studied LPA receptor. Its overexpression is associated with a poor progression-free as well as overall survival 16, and appears to be the main receptor for ovarian cancer metastatic process 17. Intriguingly, LPAR1 and LPAR2 can act in concert to promote tumor cell migration 18 or exert opposing functions, as for instance in pancreatic cancer cells 19. Taking this information together, the LPA generating enzymes and LPARs represent drug targets with a high therapeutic potential 13, 20-23.

Abundance of LPA is strongly elevated in ascites (relative to plasma) and is associated with disease progression and a poor clinical outcome 12. These findings are consistent with earlier publications reporting LPA-mediated promotion of cancer cell survival 14, 24, 25, adhesion 26, migration and/or invasion 27-32, metabolism 33 and angiogenesis 34, 35. A number of LPA-induced target genes coding for secreted proteins with functions in tumor progression have been identified in cell lines from different cancer entities, including *FGF1, IL6, CXCL8, PTGS2, VEGF-C* and *WNT11* 12, 36-38. LPA also contributes to drug resistance by maintaining OC stem cells 39 and interfering with the type I interferon response 40. LPA has further been shown to trigger cell-in-cell invasion (entosis) in tumor cells 41, which may, in an entity-dependent manner, have either pro-tumorigenic or anti-tumorigenic effects in different entities 42-46. In HGSC specifically, a role for entosis has yet to be explored.

Despite the clear association of LPA with HGSC progression and survival, the underlying LPA-regulated signal transduction network and its connections to cancer-relevant functions remain largely obscure. LPA-mediated signal transduction has previously been analyzed in A498 kidney carcinoma cells by phosphoproteomics 47. That study, however, did not yield significant novel insights, as it was restricted to identification of a relatively small number (n = 120) of up- or downregulated phosphoproteins, which were mainly associated with MAPK and ERBB signaling, not validated by follow-up validations nor experimentally linked to biological function. In the present study, we applied unbiased mass spectrometry-based phosphoproteomics to identify LPA-triggered signaling pathways in tumor cells from a HGSC patient (OCMI91s), and confirmed the data by biochemical assays and comparison with the established HGSC cell line OVCAR8. These analyses were supplemented by the identification of LPA target genes and their upstream regulators in patient-derived cells using RNA sequencing (RNA-Seq). The knowledge gained from these studies was used to mechanistically link specific signaling pathways to LPA-regulated processes, including actomyosin dynamics, cell migration and entosis.

## Materials and Methods

### Cell culture of primary OCMI cells and OVCAR cell lines

Ascites was collected from untreated patients with HGSC prior to surgery at Marburg University Hospital. The collection and analysis of human material were approved by the ethics committee at Philipps University Marburg (reference number 205/10). Donors provided their written consent in accordance with the Declaration of Helsinki. Primary tumor cell cultures (termed OCMI tumor cells) were established from ascites tumor spheroids 48 with modifications as previously described 12. This culture system allows for the propagation of HGSC cells without culture-induced crisis or genetic alterations. In the present study, we used tumor cells from patient 91 (63 years old, histological grading G3, established from spheroids < 30 µm), referred to as OCMI91s, with a maximum number of passages of 20.

The HGSC cell line OVCAR8 was obtained from the NIGMS Human Genetic Cell Repository of the NIH and cultured in RPMI 1640 (61870044, Thermo Fisher Scientific, Darmstadt, Germany) complemented with 10% FBS (FBS-LE-12A/RES1822, Capricorn Scientific, Ebsdorfergrund, Germany) and 1% Penicillin-Streptomycin (P0781, Sigma-Aldrich, Taufkirchen, Germany). Cells were routinely tested for mycoplasma contaminations.

### Reagents

LPA 16:0 (857123P), 18:0 (857128P) and 18:1 (857130P) was obtained from Avanti Polar Lipids (Alabaster, AL, USA), LPA 18:2 (L-0182) and 20:4 (L-0204) from Echelon Bioscience (Salt Lake City, UT, USA). Ro6842262 (5913), H2L5186303 (4878) were purchased from Tocris Biosciences (Bristol, UK); Y27632 (10005583), HA1077 (10010559), RKI1447 (16278), Gö6983 (13311), BIRB796 (Doramapimod, 104640), from Cayman Chemicals (Ann Arbor, MI, USA); CRT0066101 (SML1507) from Sigma-Aldrich (Taufkirchen, Germany); U0126 (9903) from Cell Signaling (Frankfurt, Germany). All inhibitors, their targets and the concentrations used are listed in Table 1.

### Antibodies

The following antibodies were used for immunoblotting and immunofluorescence staining: p-p38MAPK (T180/Y182) (4511, Cell Signaling, Frankfurt, Germany), p38MAPK (9228, Cell Signaling, Frankfurt, Germany), p-ERK1/2 (T202/Y204) (4370, Cell Signaling, Frankfurt, Germany), ERK1/2 (9107, Cell Signaling, Frankfurt, Germany), p-HSP27 (S82) (sc-166693, Santa Cruz Biotechnology, Heidelberg, Germany), HSP27 (ADI-SPA-803, Enzo Life Sciences, Lörrach, Germany), p-MYPT1 (S507) (3040, Cell Signaling, Frankfurt, Germany), p-MYPT1 (T696) (5163, Cell Signaling, Frankfurt, Germany), p-MYPT1 (T853) (4563, Cell Signaling, Frankfurt, Germany), MYPT1 (sc-514261, Santa Cruz Biotechnology, Heidelberg, Germany), p-MLC2 (T18/S19) (3674, Cell Signaling, Frankfurt, Germany), MLC2 (sc-517244, Santa Cruz Biotechnology, Heidelberg, Germany), p-LIMK1/2 (T508/T505) (3841, Cell Signaling, Frankfurt, Germany), LIMK2 (sc-365414, Santa Cruz Biotechnology, Heidelberg, Germany), p-AKT S473 (4060, Cell Signaling, Frankfurt, Germany), AKT (pan) (2920, Cell Signaling, Frankfurt, Germany), p-PKD1 (S916) (2051, Cell Signaling, Frankfurt, Germany), DOCK7 (sc-398888, Santa Cruz Biotechnology, Heidelberg, Germany), GAPDH (G9545, Sigma-Aldrich, Taufkirchen, Germany), p-PKCδ (Y311) (2055, Cell Signaling, Frankfurt, Germany), p-PAK1/2 (T423/402) (2601, Cell Signaling, Frankfurt, Germany), LDH (sc-33781, Santa Cruz Biotechnology, Heidelberg, Germany).

### Stimulation with LPA and treatment with inhibitors

The cells were serum-starved for 24 - 30 h for every assay (except for entosis assay) before stimulation with 5 µM LPA or EtOH as solvent control. LPA consisted of 1 μM 16:0 (20%), 0.25 μM 18:0 (5%), 0.5 μM 18:1 (10%), 1.625 μM 18:2 (32.5%) and 1.625 μM (32.5%) 20:4 LPA, mimicking the concentrations in ovarian cancer ascites 12. If inhibitors were used, they were added to the cells 30 min prior to the stimulation.

### RNA Sequencing

Total RNA was isolated using the NucleoSpin RNA mini kit (740955, Macherey-Nagel, Düren, Germany). RNA-Seq was carried out on an Illumina NextSeq 550 using “QuantSeq 3′ mRNA-Seq Library Prep Kit FWD for Illumina” (Lexogen, Vienna, Austria) for library preparation. Data were deposited at EBI ArrayExpress (accession number E-MTAB-12487) and processed as described previously 49 using Ensembl 96 50. Data are presented as 'counts per million' (CPM).

### Proteomic and phosphoproteomics analyses

For phosphoproteomic analyses, cells were treated with LPA, antagonists or solvent at least in triplicate as described above and lysed in 100 mM Tris pH 7.6, 4% SDS, PhosSTOP (4906845001, Roche, Basel, Switzerland), Protease Inhibitor Cocktail (P8340, Sigma Aldrich, Taufkirchen, Germany). Analysis of OCMI91s lysates were performed as described 51. The protocol was modified to use 125 μg of peptide per channel for TMT labeling, resulting in 500 μg multiplexed samples. Analysis of OVCAR8 lysates proceeded as follows: samples were subjected to acetone precipitation and following tryptic digest of 600 μg total protein per sample as described 51, as well as desalting using Waters Oasis HLB cartridges (186006339, Waters, Eschborn, Germany) and peptide yield determination using the Quantitative Fluorometric Peptide Assay (23290, Thermo Fisher Scientific, Darmstadt, Germany), each sample was labeled using TMTsixplex (90064, Thermo Fisher Scientific, Darmstadt, Germany) following the manufacturer's protocol. Subsequent to mass spectrometric validation of labeling efficiency and mixing 80 μg peptides per channel using samples from solvent control experiments as bridging samples where more than a single sixplex was required to cover replicate experiments, multiplexed samples were desalted once more as described above. Of the resulting pooled samples, 30 μg each were fractionated into eight fractions using High pH Reversed-Phase Peptide Fractionation Kit (84868, Thermo Fisher Scientific, Darmstadt, Germany) using the manufacturer's protocol and evaporation to dryness for subsequent analysis of the background proteome. In the remaining multiplexed peptide samples, phosphorylated peptides where enriched using the High-Select Fe-NTA Phosphopeptide Enrichment Kit (A32992, Thermo Fisher Scientific, Darmstadt, Germany). While 5% (10 μl) of the resulting eluate where evaporated to dryness for direct subsequent liquid chromatography/tandem mass spectrometry (LC-MS^2^) analysis, the remainder was once more separated into eight fractions using the High pH Reversed-Phase Peptide Fractionation Kit and fractions subsequently evaporated to dryness.

LC-MS2 analysis was performed on 50% of the corresponding sample material rehydrated in 0.1% formic acid as published 51. Detailed information on instrumentation parametrization was extracted and summarized using MARMoSET 52 and is included in the repository-deposited data sets. Peptide/spectrum matching as well as TMT quantitation was performed using the MaxQuant suit of algorithms (versions 1.6.8.0 & 2.0.3.0 for OCMI91s and OVCAR8 data sets, respectively 52 against the human canonical and isoforms Uniprot database (OCMI91s: downloaded 20190819, 173199 entries; OVCAR8: downloaded 20211026, 202160 entries). Mass spectrometric raw data along with documentation of instrumentation parameters governing its acquisition as well as MaxQuant parameters employed are available at the Center for Computational Mass Spectrometry's (CCMS) MassIVE data repository (a full member of the ProteomeXchange consortium) 53 with identifiers MSV000091146 and MSV000091158.

Data were filtered for rows containing no zero values, and analyzed by paired Student's t-test (blocking on replicate set / phosphoproteome analysis chip). Thresholds were set according to the dynamic range of the assay to FC > 1.2x or < 0.83x and p < 0.1.

### Functional annotations

The following tools and databases were used for functional annotations of genes and proteins: kinome database 54; curated transcription factor database 55; Gene Ontology (GO) Biological Process Complete 56, Reactome Pathways 57 and the Ingenuity Pathway Analysis (IPA) database (content version: 26127183, 2015-11-30; QIAGEN Inc.) with Python-based search functions and statistical analysis tools.

### Immunoblotting and quantification

The cells were lysed in the same lysis buffer used for phosphoproteome analysis containing 100 mM Tris-HCl (pH 7.6), 4% SDS (in PBS), Protease Inhibitor Cocktail (P8340, Sigma-Aldrich, Taufkirchen, Germany) and phosphatase inhibitor mix (10 mM β-glycerophosphate, 1 mM sodium orthovanadate, 10 mM sodium fluoride and 10 mM sodium pyrophosphate). Immunoblots were performed according to standard immunoblotting protocols. To avoid stripping and reprobing when analyzing phosphoproteins, multiplex fluorescent western blotting was performed using the Immobilon-FL PVDF membrane (IPFL00005, Merck Millipore, Darmstadt, Germany) and α-rabbit IRDye® 800CW (926-32211, LI-COR, Bad Homburg, Germany) and α-mouse IRDye® 680RD (926-68070, LI-COR, Bad Homburg, Germany) secondary antibodies. Phosphoprotein signals were normalized against the respective total protein signal. As we weren't able to obtain a reliable PKD1 antibody from mouse, p-PKD1 was normalized against GAPDH. Imaging and quantification were carried out using the ChemiDoc MP system and Image Lab software version 6.1 (Bio-Rad, Feldkirchen, Germany).

### Measurement of intracellular Ca^2+^ release

Calcium assays were carried out in 96-well black ViewPlate (6005182, PerkinElmer, Rodgau, Germany) using a Fura-2 Calcium Flux Assay Kit (ab176766, Abcam, Cambridge, UK) according to the instructions of the manufacturer. Briefly, the cells were pre-loaded with Fura-2 AM for 60 min at 37°C followed by 20 min incubation time at RT. Kinetic measurements were carried out at least in technical triplicates on a VICTOR® Nivo™ Microplate Reader (PerkinElmer, Rodgau, Germany), measuring at 340 and 380 nm after every 4 sec for 25 cycles. After 4 cycles of background measurement, 5 µM LPA or solvent was added to the cells. The 340/380 nm ratio before each treatment was used as reference for normalization and the area under the curve (AUC) was calculated from the Fura-2 trace.

### Measurement of intracellular cAMP

For the quantitative determination of cyclic AMP (cAMP) in the cell lysate, cAMP Parameter Assay Kit (KGE002B, R&D Systems, Minneapolis, MN, USA) was used according to the manufacturer's protocol. The cAMP phosphodiesterase inhibitor IBMX (13347, Cayman Chemicals, Ann Arbor, MI, USA) was added to the cells at a concentration of 0.1 mM for 15 min prior to stimulation with 5 µM LPA or solvent for another 10 min. The OD was measured with SpectraMax 340 microplate reader (MWG Biotech, Ebersberg, Germany) in technical duplicates at 450 nm with wavelength correction measured at 570 nm. The cAMP concentration was calculated according to the standard curve generated using four parameter logistic (4-PL) curve-fit.

### RT-qPCR

Total RNA was isolated using the NucleoSpin RNA mini kit (740955, Macherey-Nagel, Düren, Germany), cDNA synthetized with iScript cDNA Synthesis Kit (1708891BUN, BioRad, Feldkirchen, Germany) and RT-qPCR performed in technical triplicates with ABsolute SYBR Green Mix (AB-1158B, Thermo Fisher Scientific, Darmstadt, Germany) according to the instructions of the manufacturers using Stratagene Mx3000P qPCR System (Agilent Technologies, Waldbronn, Germany). Results were evaluated by the Cy0 method 58 with *RPL27* used for normalization. The following primers were used:

*RPL27*: AAAGCTGTCATCGTGAAGAAC and GCTGTCACTTTGCGGGGGTAG;

*IL6*: AGGAACAAGCCAGAGCTGTGCAGATG and TTTGTGGTTGGGTCAGGGGTGGTTA; THBS1: TCTCTGACCTGAAATACGAATGTAG and AAGGAAGCCAAGGAGAAGTG; CCL20: GCTGCTTTGATGTCAGTGCT and GCAGTCAAAGTTGCTTGCTTC;

*LPAR1*: ATTTCACAGCCCCAGTTCACA and ACCAGCTTGCTGACTGTGTT;

*LPAR2*: GCCTGGTCAAGACTGTTGTCA and CCAGGACATTGCAGGACTCA;

*LPAR3*: CCAACGTCTTGTCTCCGCATA and CCGGGGTCCAGCATACCA

### Immunofluorescence

The cells were plated on a Ø12 mm glass coverslip, stimulated with LPA or solvent for 1 h, fixed with 4% formaldehyde (in PBS) for 10 min at RT, permeabilized with 0.3% Triton X100 for 5 min and blocked with 10% goat serum (G9023, Sigma-Aldrich, Taufkirchen, Germany) (in PBS) for 60 min at RT. The cells were incubated in humid chamber over night at 4°C with primary antibodies and incubated on the next day with following secondary antibodies for 2 h at RT: a-rabbit AF488 (A11008, Thermo Fisher Scientific, Darmstadt, Germany) and a-mouse AF488 (A11029, Thermo Fisher Scientific, Darmstadt, Germany). F-actin was stained with Phalloidin conjugated to California Red (ABD-23103, AAT Bioquest, Pleastanon, CA, USA) for 30 min at RT followed by DNA staining with 10 µg/ml Hoechst 33342 (B2261, Sigma-Aldrich, Taufkirchen, Germany) for 15 min at RT. The coverslips were mounted onto the microscope slides with a drop of VECTASHIELD® Antifade Mounting Medium (H-1000-10, Vector Laboratories, Newark, CA, USA) and images were taken using the confocal microscope Leica SP8i (Leica Microsystems, Wetzlar, Germany) using Leica Application Suite X (LASX) 3.5.7.23325.

### Wound closure assay

The cells were grown in silicone Culture-Inserts 4 Well (80469, Ibidi, Gräfelfing, Germany) to create the cell-free area (wound). After reaching confluence, the inserts were removed and cells washed with PBS before preincubation with the inhibitors for 30 min and stimulated with LPA or EtOH. Pictures were taken at a 10x magnification from four different fixed areas with a Leica DMI3000B microscope (Leica Microsystems, Wetzlar, Germany) using Leica Application Suite (LAS) V4.7 at time points 0 and 8 h. The cell-free area was analyzed and calculated using ImageJ (NIH) software.

### RNA interference

Small interfering RNA (siRNA) transfection was performed according to the manufacturer's protocol using the TransIT-X2 reagent (MIR-6000, Mirus Bio, Madison, WI, USA) for OCMI91s cells or HiPerFect transfection reagent (301705, Qiagen, Hilden, Germany) for OVCAR8 cells. For the knock down of LPARs, a second transfection was performed after 48 h. Following siRNA oligonucleotides were used (all from Sigma-Aldrich, Taufkirchen, Germany): MYPT1: SASI_Hs01_0003116; DOCK7: SASI_Hs01_00234094, SASI_Hs01_00234093, SASI_Hs01_00234095; LPAR1: SASI_Hs01_00172897, SASI_Hs01_00172900, SASI_Hs01_00172898, SASI_Hs01_00172899; LPAR2: SASI_Hs01_00175376, SASI_Hs01_00175378, SASI_Hs01_00175377; LPAR3: SASI_Hs02_00343735, SASI_Hs01_00036121, SASI_Hs01_00036117. For DOCK7 and LPARs, equimolar mixtures of the siRNA oligonucleotides were used. MISSION siRNA Universal Negative Control #1 (SIC001, Sigma-Aldrich, Taufkirchen, Germany) was used as a control. The cells were harvested 48 - 72 h after transfection.

### Transwell migration assay

Falcon transwell inserts with 0.8 µm pore size (353097, Corning, Corning, NY, USA) were used for the migration assays. The inserts were equilibrated for 1 h in the cell culture incubator at 37°C in the respective media without FCS in the upper compartment (OCMI media for OCMI91s, RPMI1640 for OVCAR8) and media with FCS as chemoattractant in the lower compartment (5% for OCMI91s, 10% for OVCAR8). After equilibration, the cells were added to the upper compartment and let migrate for 8 h (OCMI91s) or 24 h (OVCAR8) in the cell culture incubator at 37°C. After removing non-migrated cells from the upper compartment with a cotton swab, the migrated cells were fixed with 4% formaldehyde (in PBS) for 10 min at RT and stained with 10 µg/ml Hoechst33342 (B2261, Sigma-Aldrich, Taufkirchen, Germany) for 15 min at RT. Pictures were taken from five different areas of the membrane with a Leica DMI3000B microscope (Leica Microsystems, Wetzlar, Germany) using Leica Application Suite (LAS) V4.7 and the migrated cells counted with ImageJ (NIH) software.

### Entosis assay

Cells were plated on a Corning® Costar® Ultra-Low Attachment 24 Well Plate (CLS3473, Corning, Corning, NY, USA) and stimulated with 15% FCS, 5 µM LPA or EtOH. After 4 h, the cells were fixed directly in suspension with 4% formaldehyde (in PBS) for 10 min, washed with PBS and dried onto Poly-L-Lysine (P8920, Sigma-Aldrich, Taufkirchen, Germany) coated Ø12 mm glass coverslips in a hybridization oven (Mini Oven MK II, MWG Biotech, Ebersberg, Germany) for 45 - 60 min at 60°C. The cells were then permeabilized with 0.5% Triton-X100 (in PBS) for 10 min at RT and incubated for 30 min at RT or overnight at 4°C with Phalloidin AF488 (ABD-23153, AAT Bioquest, Pleastanon, CA, USA). If additional proteins were stained (according to the immunofluorescence protocol described before), Phalloidin conjugated to Californian Red was used. DNA was stained with 10 µg/ml Hoechst33342 (B2261, Sigma-Aldrich, Taufkirchen, Germany) for 15 min at RT. The coverslips were mounted onto the microscope slides with a drop of VECTASHIELD® Antifade Mounting Medium (H-1000-10, Vector Laboratories, Newark, CA, USA) and images were taken using the confocal microscope Leica SP8i (Leica Microsystems, Wetzlar, Germany) using Leica Application Suite X (LASX) 3.5.7.23325. Images were analyzed with ImarisViewer 9.8.0 software (Oxford Instruments, Wiesbaden, Germany) and ImageJ (NIH) was used to count entotic events and total cells.

### Histochemical staining of HGSC spheroids

Tumor cell spheroids were obtained from ascites by consecutive filtration using 30 and 100 µm cell strainers (130-098-458 and 130-098-463, Miltenyi Biotech, Bergisch Gladbach, Germany). The cells were fixed with 4% formaldehyde, embedded into 2% agarose and processed into paraffin blocks. 4 µm microtome cuts of samples were taken and stained with hematoxylin (T865.2, Carl Roth, Karlsruhe, Germany) according to standard protocols.

### Statistical analysis

Comparative RT-qPCR, immunoblot and immunofluorescence data were statistically analyzed by paired Student's t test (two-sided, equal variance) unless indicated otherwise. Significance levels are indicated as ****, ***, ** and * for p < 0.0001, p < 0.001, p < 0.01 and p < 0.05, respectively. The statistical significance of OCMI91s/OVCAR8 phosphosite overlap was assessed by hypergeometric test based on the total number of observed proteins with phosphosites in both datasets, the number of proteins with at least one differential phosphosite in each dataset, and their overlap.

## Results

### LPA target genes in patient-derived HGSC cells

To date, the identification of LPA target genes is limited to established tumor cell lines 12, 36-38. To better reflect the *in vivo* situation, we determined the transcriptional profile of patient-derived OCMI91s cells (see Material and Methods for details) to identify LPA-regulated genes. Cells were stimulated with an LPA mixture approximating the LPA composition in ascites (subsequently referred to as LPA) with and without the LPAR antagonists Ro6842262 (Ro; LPAR1-selective) or H2L5186303 (H2L; LPAR2-selective). RNA-Seq analysis of these samples (Table S1) identified n = 128 genes upregulated by LPA (FC > 2-fold; CPM > 2; inhibition by Ro or H2L > 50%; Table S2) and n = 67 genes downregulated by LPA (FC < 0.5-fold; CPM > 2; inhibition by Ro or H2L > 50%; Table S3). Of the 128 upregulated genes n = 112 (87.5%) were sensitive to Ro, and n = 55 of the n = 67 (82.1%) downregulated genes, indicating a major role for LPAR1 in regulating transcription.

**Figure 1:**
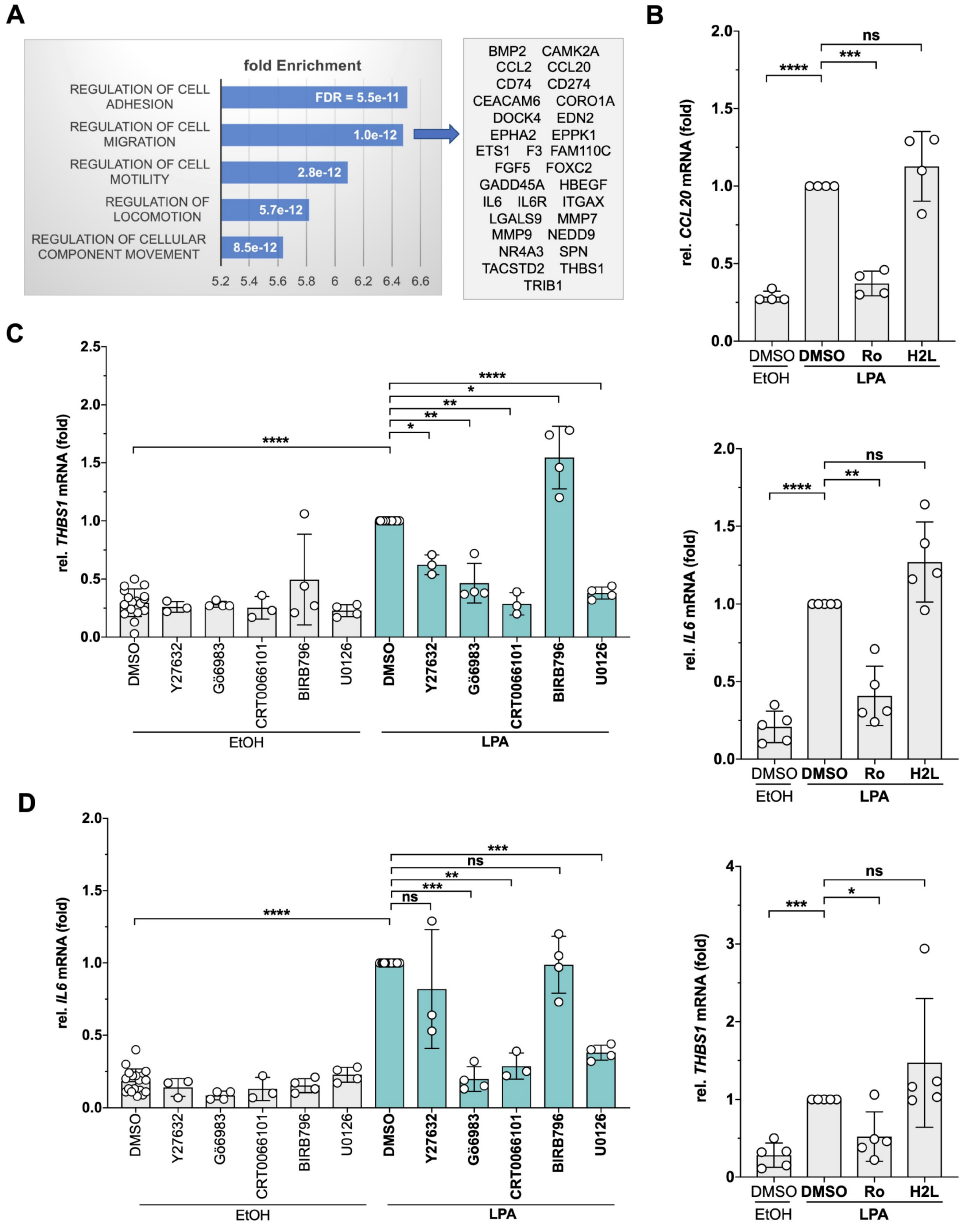
** Identification of LPA-induced genes and effect of pharmacological inhibition of signal transduction components. (A)** OCMI91s cells were treated with 1 µM Ro6842262, 1 µM H2L5186303 or DMSO before stimulation with 5 µM LPA mix or EtOH for 2 h. RNA was analyzed by RNA-Seq (Table S1). Genes upregulated by LPA ( > 2-fold; CPM > 2; inhibition by Ro6842262 > 50%; n = 112) were analyzed for enrichment of GO terms (Table S4). The plot shows the fold enrichment and FDR for the top 5 terms. Genes enriched for migration (n = 31; Table S5) are listed on the right-hand side. **(B)** Verification of the RNA-Seq data by RT-qPCR for *THBS1*, *IL6* and *CCL20* (n = 4-5 biological replicates). **(C, D)** Effect of selective protein kinase inhibitors on *THBS1* and *IL6* mRNA expression. OCMI91s cells were treated with Y27632, Gö6983, BIRB796, U0126 (all 10 µM), CRT0066101 (2.5 µM) or DMSO (solvent) before stimulation with 5 µM LPA mix or EtOH for 2 h. *THBS1* and *IL6* mRNA was analyzed by RT-qPCR (n = 3-4 biological replicates). Plots depict the mean ± SD. Asterisks indicate p values determined by two-sided, paired t-test: **** p < 0.0001; *** p < 0.001; ** p < 0.01; * p < 0.05; ns: not significant.

Gene ontology (GO) enrichment analysis of the Ro-sensitive upregulated genes identified 'cell adhesion', 'migration', 'motility' and 'locomotion' as the top terms (Table S4; bar diagram in Fig. 1A). The genes representing the term 'migration' (Table S5; Fig. 1A, right panel) encompass cytokines, membrane receptors, metalloproteases as well as cytoplasmic and nuclear signal transduction proteins with known pro-tumorigenic functions, pointing to a complex transcriptional response to LPA in HGSC cells. LPA-mediated induction of 3 genes (*CCL20*, *IL6*, *THBS1*) was verified by RT-qPCR (Fig. 1B). Consistent with the RNA-Seq data, LPA induction was sensitive to Ro, but not to H2L.

To gain insight into the signal transduction mechanisms triggered by LPA in patient-derived OCMI91s cells, we performed Ingenuity Pathway Analysis of LPA-induced, Ro-sensitive genes. As shown in Table S6, multiple pathways are predicted to transduce LPA signals to target genes, including ERK, JNK, p38, PKC, RAF, AKT and CREB, which is in agreement with observations in other systems 59. To test and extent these predictions, we analyzed the effects of protein kinase inhibitors selective for MEK1/2 (upstream kinase of ERK), p38, different PKC isoforms (PKCα/β/δ/γ/ζ), PKD1-3 and ROCK1/2 (Table 1) on *THBS1* and *IL6* expression. As illustrated in Fig. 1C, all tested inhibitors significantly diminished the LPA-mediated induction of *THBS1* - except for the p38 inhibitor BIRB796 which enhanced expression. Similarly, Gö6983 (PKC inhibitor), CRT0066101 (PKD inhibitor) and U0126 (ERK1/2 inhibitor) significantly inhibited *IL6* expression (Fig. 1D). Our findings point to a complex LPA signaling network in patient-derived HGSC cells that we set out to dissect by the phosphoproteomics.

### Phosphoproteomic analysis of LPA-induced signal transduction

**Figure 2:**
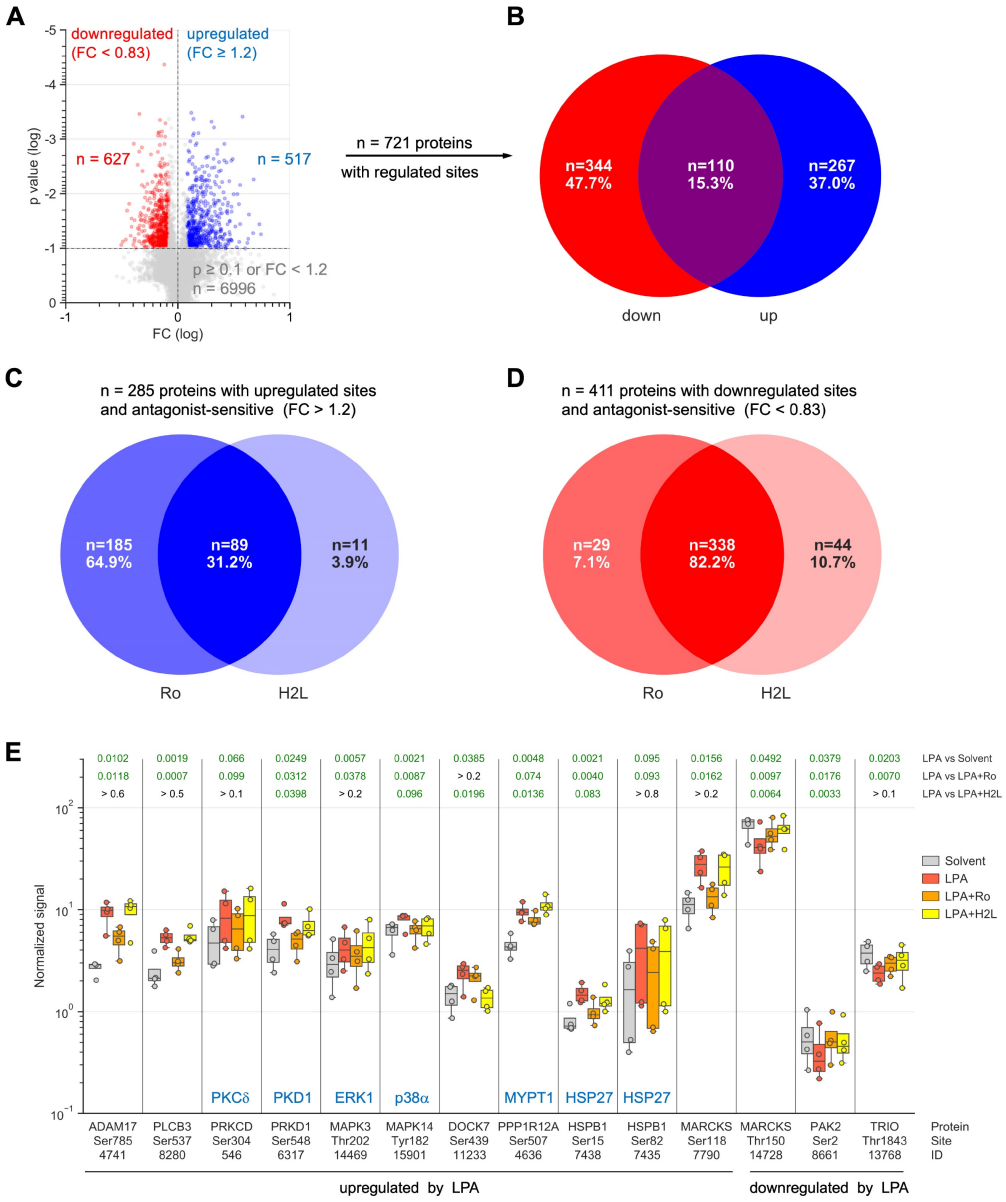
** Effect of LPA and LPAR inhibitors on the phosphoproteome analysis of OCMI91s cells.** Cells were treated with 1 µM Ro, 1 µM H2L or DMSO (solvent) followed by stimulation with 5 µM LPA or EtOH (solvent) for 15 min. **(A)** Volcano plot showing phosphosites regulated by LPA. Blue: upregulated sites (FC > 1.2 and p < 0.1). Red: downregulated sites (FC < 0.83 and p < 0.1). Grey: sites not significantly affected or FC below threshold. **(B)** Venn diagram showing the overlap of proteins with upregulated and/or down-regulated phosphosites from panel A. **(C, D)** Venn diagrams illustrating the effect of LPAR antagonists on proteins with upregulated (C) or down-regulated (D) phosphosites. **(E)** Examples of LPA-regulated phosphorylation sites and effects of Ro and H2L. Figures show the median (line), upper and lower quartiles (box), range (whiskers) and values of n = 4 replicates (circles). Significance was tested by paired t test; p values are shown at the top. Green numbers indicate p < 0.1.

To gain deeper insight into the LPA-regulated signaling pathways, OCMI91s cells were treated with Ro, H2L or solvent (DMSO) followed by stimulation with LPA or solvent (EtOH) for 15 min and analyzed by MS-based phosphoproteomics, which identified 7.202 phosphosites associated with known genes (Table S7). After normalization to total proteome signals, we found n = 517 upregulated sites (Fig. 2A) in n = 377 proteins (Fig. 2B; Table S8; ≥ 3 evaluable replicates) and n = 627 downregulated sites (Fig. 2A) in n = 454 proteins (Fig. 2B; Table S9). Notably, 15% (n = 110) of these proteins contained both upregulated and downregulated phosphosites (Fig. 2B).

Inspection of the upregulated sites revealed a prominent role for LPAR1 (Fig. 2C), as the percentage of proteins selectively affected by Ro (65%) was much larger than selective effects of H2L (4%). Inhibition by both antagonists amounted to 31%, suggesting that a large fraction of phosphorylated targets depend on both LPAR1 and LPAR2. For downregulated sites, we found Ro or H2L selectivity for 7% and 11% of the affected proteins, respectively, and inhibition by both antagonists for 82% (Fig. 2D), indicating that both receptors figure in the downregulation of the majority of phosphosites. This observation is relevant in view of the high expression of *LPAR1* compared to *LPAR2* in OCMI91s cells (Fig. S1), and suggests that the expression levels of LPARs may have little effect on their signaling function.

Functional annotation of the 377 proteins with LPA-upregulated phosphosites revealed a strong association with 'cytoskeleton organization' (n = 78; GO enrichment analysis; Tables 2 and S10) as the most significant biological process. We also interrogated the Ingenuity Pathway Analysis database for association with 'migration' or 'metastasis' and found n = 100 hits (Table 2; Fig. S2). Reactome pathway analyzes further showed that signaling components constitute a large subgroup of LPA targets with RHO-GTPase signaling clearly the most significantly affected pathway (n = 57 proteins; Tables 2 and S11; Reactome pathway). Moreover, n = 25 protein kinases and n = 17 transcription factors are found among the proteins with upregulated phosphosites (Table 2; Fig. S3). Similar associations were observed for proteins with downregulated phosphosites (Tables 3, S12 and S13; Fig. S4): cytoskeleton organization' (n = 87), 'migration' or 'metastasis' (n = 108), RHO-GTPase signaling (n = 71), protein kinases (n = 19) and transcription factors (n = 19).

LPA-mediated regulation of several proteins with key functions in signal transduction and metastasis-associated processes is illustrated in detail in Fig. 2E. These include examples of proteins with up- and downregulated phosphosites (e.g., MARCKS) and antagonist-selective effects (e.g., ADAM17: Ro-selective inhibition; DOCK7: H2L-selective inhibition). Taken together, our data point to a complex network of both stimulatory and inhibitory phosphosites that are up- or downregulated by LPA via LPAR1 and/or LPAR2.

### Verification of LPA-regulated phosphoproteins and analysis of second messengers

We next sought to verify the phosphoproteomic data for various LPA-triggered signal transduction pathways. To this end, we used phosphosite-specific antibodies directed at the sites identified by MS (p38, HSP27, MYPT1, ERK1/2), or, where not available, antibodies to other known phosphosites with presumed similar regulatory functions (PKD1, LIMK2).

**Figure 3:**
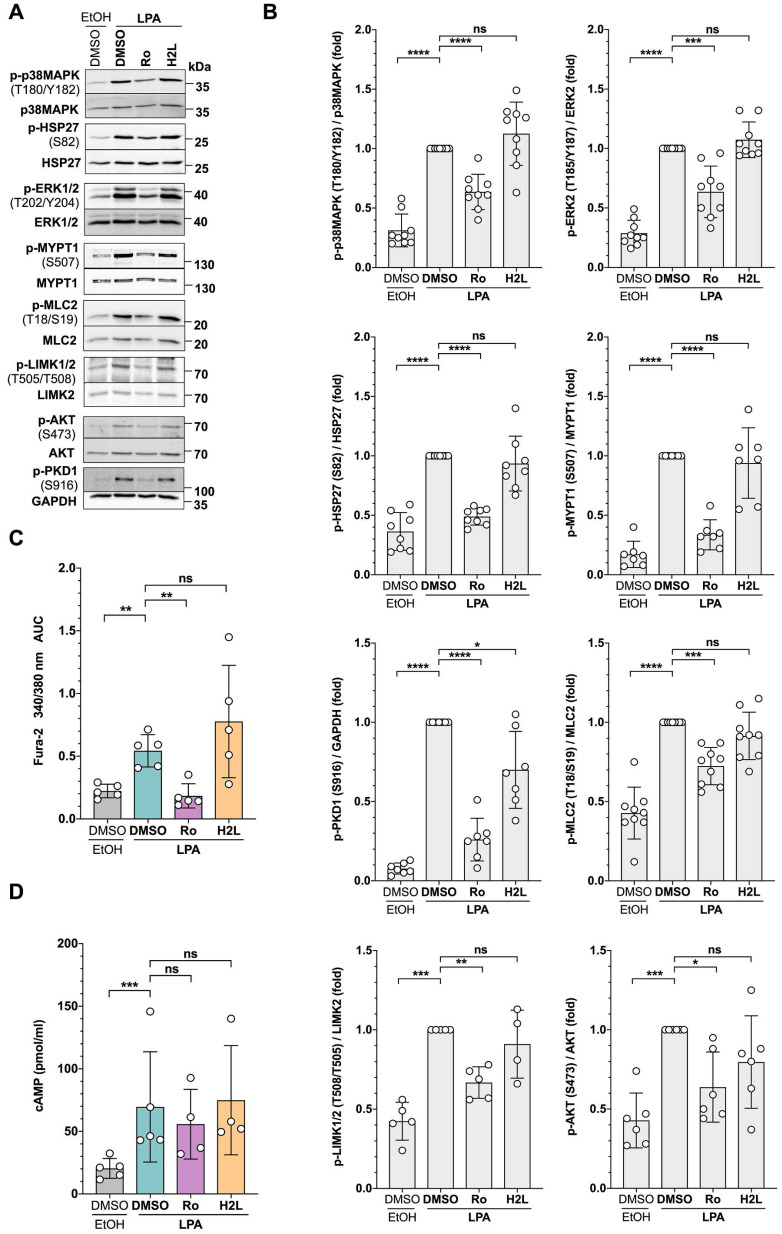
** Validation of signaling pathways identified by phosphoproteomics in OCMI91s cells. (A)** Representative immunoblots of phosphoproteins after treatment with LPA ± LPAR antagonists. Cells were treated with 1 µM Ro, 1 µM H2L or solvent (DMSO) prior to stimulation with 5 µM LPA or solvent (EtOH) for 5 min. Blots were probed with the phosphosite-specific antibodies and the corresponding protein-directed antibodies or antibodies to GAPDH as loading controls. **(B)** Quantification of n = 4-9 biological replicates showing that LPA-induced phosphorylation is majorly mediated by LPAR1. **(C)** Increase of intracellular Ca^2+^ after LPA stimulation. OCMI91s cells were loaded with Fura-2 and treated with 1 µM Ro, 1 µM H2L or solvent (DMSO) before stimulation with 5 µM LPA or solvent (EtOH). Kinetic measurements of Fura-2 fluorescence are presented as AUC of Fura-2 trace (see Materials and Methods for details). **(D)** Increase of cAMP levels in cell lysates after preincubation with 1 µM Ro, 1 µM H2L or solvent (DMSO) and stimulation with 5 µM LPA or solvent (EtOH) for 10 min measured by competitive enzyme immunoassay. Each dot represents a biological replicate. Shown is the mean ± SD. Asterisks indicate p values determined by two-sided, paired t-test: **** p < 0.0001; *** p < 0.001; ** p < 0.01; * p < 0.05; ns: not significant.

In addition, we included antibodies to phospho-MCL2 and phospho-AKT as indicators of activated ROCK and Gi/o signaling, respectively 15. As shown in Fig. 3A and 3B, immunoblotting confirmed the LPA-induced phosphorylation found using phosphoproteomics at T202/Y204 of ERK2 (MAPK1), T180/Y182 of p38 (MAPK14), S82 of HSP27 (HSPB1), and S507 of MYPT1 (PPP1R12A). MYPT1 is a stimulatory subunit of protein phosphatase 1 (PP1), which in turn is a negative regulator of myosin light chain 2 (MLC2) 60-68. Additionally, immunoblotting found LPA-induced phosphorylation of S916 of PKD1 (PRKD1; S548 found my MS), S473 of AKT, T508 of LIMK2 (S314 found by MS) and T18/S19 of MLC2 (MYL9). The data also confirmed the Ro-selective inhibition of LPA-triggered phosphorylation in all cases analyzed, pointing to a selective role for LPAR1.

Consistent with the LPA-induced phosphorylation of phospholipase C β3 (PLCB3), observed in the phosphoproteome (Table S8), we found a significant increase of intracellular Ca^2+^ after LPA stimulation, which was selectively inhibited by Ro (Fig. 3C). Furthermore, and in agreement with the phosphorylation changes found for a regulatory PKA subunit (S58 of PRKAR2A), intracellular cAMP concentration was significantly elevated by LPA treatment, an effect that appeared slightly, albeit not statistically significant, inhibited by Ro (Fig. 3D).

**Figure 4:**
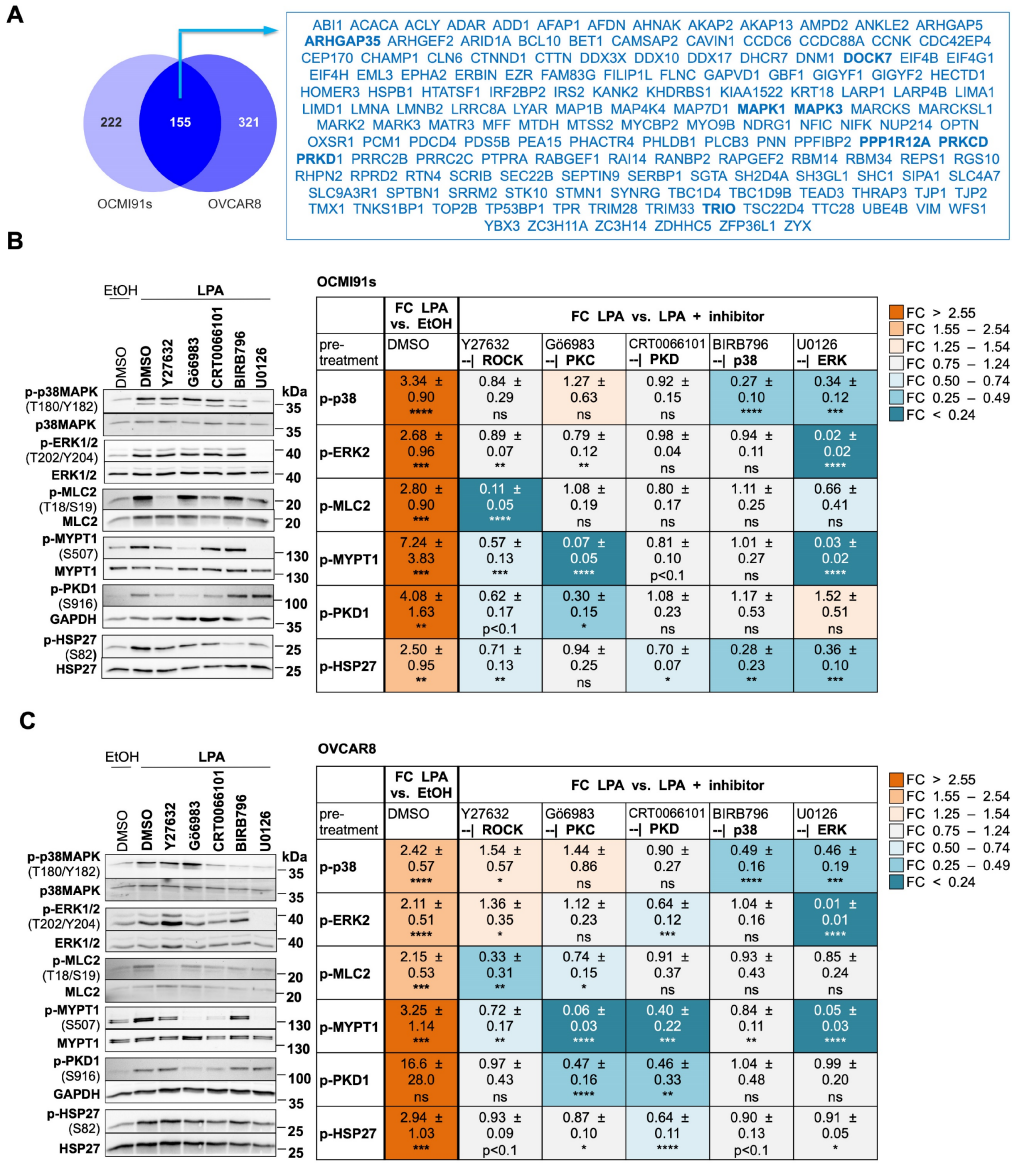
** Comparative analyses of LPA-induced signaling components in OCMI91s and OVCAR8 cells. (A)** Venn diagrams illustrating the overlap of proteins with LPA-upregulated phosphosites in OCMI91s and OVCAR8 cells (FC > 1.2; data in Tables S8 and S14). Proteins with central functions in the LPA-induced signaling network modeled in Fig. 5 are marked in bold. **(B, C)** Effects of selective protein kinase inhibitors. OCMI91s cells (B) and OVCAR8 cells (C) were treated with Y27632, Gö6983, BIRB796, U0126 (all 10 µM), CRT0066101 (2.5 µM) or solvent (DMSO) before stimulation with 5 µM LPA mix or solvent (EtOH) for 5 min. Left panels show representative immunoblots, and right panels the quantification of n = 7-11 biological replicates. Colors reflect the fold change of phosphorylation relative to solvent control (left-most column) or relative to LPA (all other columns). Values represent the mean ± SD. Asterisks indicate p values determined by two-sided, paired t-test: **** p < 0.0001; *** p < 0.001; ** p < 0.01; * p < 0.05; ns: not significant.

We next asked whether the observations made with patient-derived OCMI91s cells may be corroborated in an established HGSC cell line. Phosphoproteomic analyses of LPA-stimulated OVCAR8 cells followed by intersection with the data set from OCMI91 cells identified n = 155 proteins with phosphosites upregulated in both (Fig. 4A; Tables S8 and S14). These represent 41% and 33% of upregulated phosphoproteins in OCMI91s and OVCAR8 cells, respectively - a highly significant overlap by hypergeometric test (enrichment 3.0-fold; p = 4x10^-46^). Importantly, a majority of phosphoproteins representing central nodes in the signaling network constructed in the following section were regulated in both OCMI91 and OVCAR8 cells, including ARHGAP35, DOCK7, MAPK1 (ERK2), MAPK3 (ERK1), PKCδ, PKD1, PPP1A12A (MYPT1), as well as TRIO (boldface in Fig. 4A). For downregulated phosphosites, we also found a significant overlap of n = 53 common proteins (enrichment 3.3-fold; p = 2x10^-17^), corresponding to 12% and 29% of downregulated phosphoproteins in OCMI91s and OVCAR8 cells, respectively (Fig. S5; Tables S9 and S15). These relatively low percentages are likely due to a low number of downregulated phosphosites in OVCAR8 cells (n = 122 compared to n = 454 in OCMI91s cells). We therefore focused further studies on proteins with upregulated phosphosites.

### Effect of selective protein kinase inhibition on LPA-induced signaling components

To facilitate the integration of the identified phosphoproteins into a signaling network, we analyzed the regulation of p38, ERK2, MLC2, MYPT1, PKD1 and HSP27 by LPA in more detail. Immunoblotting confirmed that the phosphorylation of all six proteins is significantly and strongly upregulated by LPA in both OCMI91s (Fig. 4B) and OVCAR8 cells (Fig. 4C). Next, we studied the effects of selective inhibitors of ROCK, PKC, PKD, p38 and ERK1/2 (Table 1) on the LPA-induced phosphorylation of these proteins. The results indicate several regulatory features common to OCMI91s and OVCAR8:

- ROCK inhibition significantly reduced p-MLC2 and to a lesser extent of p-MYPT1;

- PKC inhibition significantly reduced p-MYPT1 and to a lesser extent of p-PKD1;

- PKD inhibition significantly reduced p-HSP27;

- ERK inhibition significantly reduced both p-p38 and p-MYPT1.

In addition, we observed OCMI91-specific inhibitory effects in some cases, i.e., for ROCK inhibition -| p-PKD and p-HSP27; for p38 inhibition -| p-HSP27; and for ERK inhibition -| p-MLC2 and p-HSP27. OVCAR8-specific inhibitory effects, were seen for PKC inhibition -| p-MLC2; for ERK inhibition -| p-MLC2; and for PKD inhibition -| p-ERK2 and p-MYPT1. Several combinations also showed stimulatory effects (PKC inhibition → p-p38 in both cell lines; ERK inhibition → p-PKD in OCMI91s cells; and ROCK inhibition -| p-p38 and p-ERK2 in OVCAR8 cells).

One of the salient features of this analysis is the upregulation of MYPT1 phosphorylation at S507 by multiple signaling pathways, as a significant reduction was observed with ROCK, PKC or ERK inhibition in both cell systems, as well as by PKD inhibition in OVCAR8 cells. Phosphorylation of its substrate MLC2 was significantly reduced by ROCK inhibition in both OCMI91s and OVCAR8 cells, but the effect of ERK inhibition on MLC2 was OCMI91-specific and, *vice versa*, the effect of PKC inhibition was OVCAR8-specific. Furthermore, ROCK inhibition affected p-MLC2 markedly stronger than p-MYPT1, while the opposite applied to PKC and ERK inhibition. These findings indicate that MLC2 phosphorylation only weakly correlates with S507 phosphorylation of MYPT1, pointing to a complex regulatory mechanism, which we address in detail further below.

### Model of an LPA-regulated signaling network

**Figure 5:**
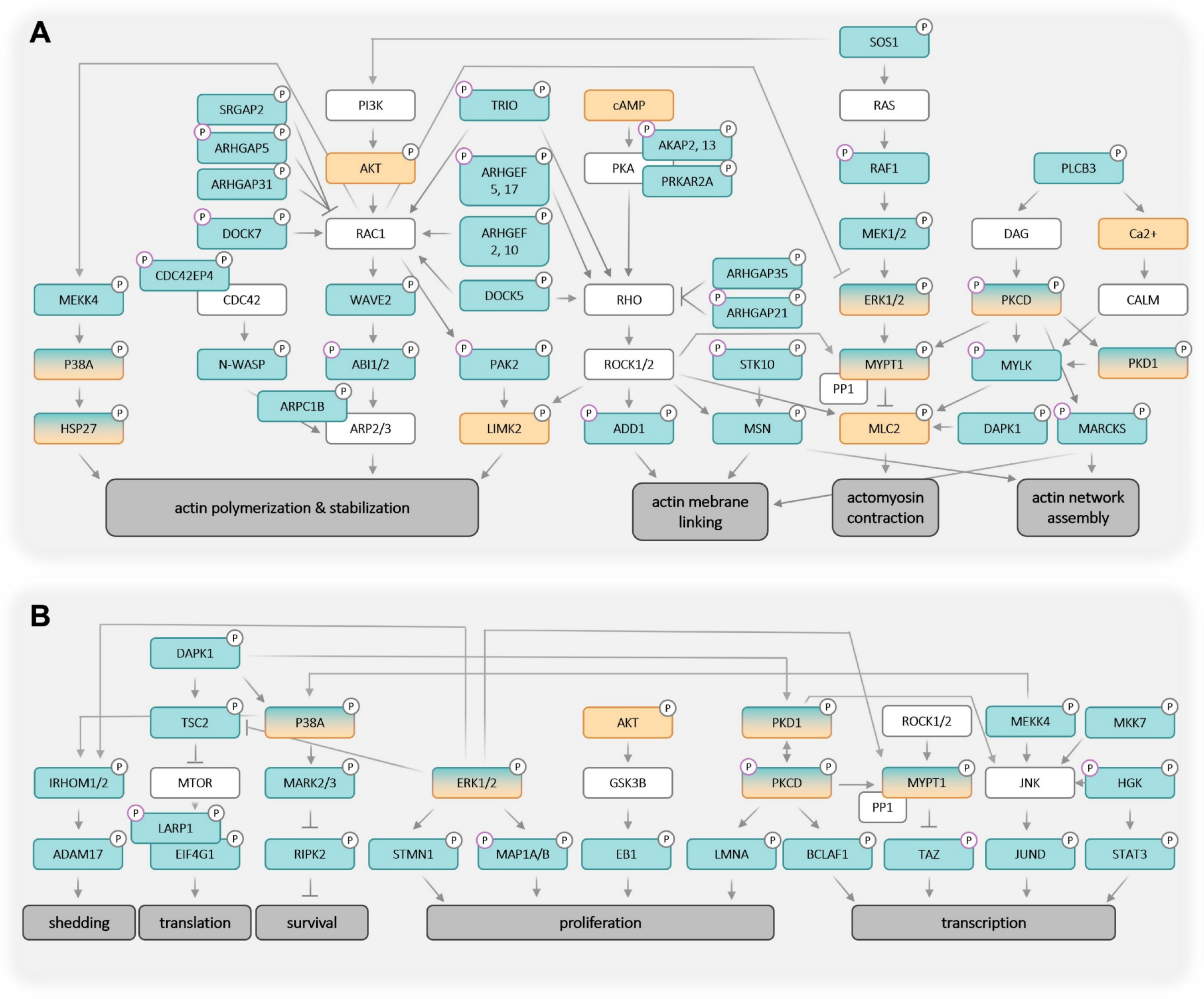
** Model of LPA-induced signaling network OCMI91s cells in the regulation of actomyosin dynamics (A) and other processes (B).** Cyan boxes: regulated proteins identified by phosphoproteomics analysis (FC > 1.2 or < 0.83; p < 0.1; Tables S8 and S9); orange boxes: regulated proteins or second messengers identified with Western Blot or other assays; green/orange boxes: regulated proteins identified by phosphoproteomics and validated with Western Blot; white boxes: missing known pathway components; upregulation of phosphorylation is marked with by grey, downregulation by a purple ring.

Integration of the identified LPA-regulated signaling proteins into pathways is illustrated in the network model in Fig. 5. It is evident that LPA-triggered signaling is linked to multiple biological processes relevant to tumorigenesis and tumor progression. Among these actomyosin dynamics stands out (Fig. 5A), as numerous components of pathways centered around RHO GTPase are targets of LPA-mediated signaling, including at least 13 GEFs and GAPs regulating RHO and RAC activities 53, e.g., AHRGEF, AHRGAP and DOCK family members:

- the RAC GEFs ARHGEF2, ARHGEF10 and DOCK7;

- the RAC GAPs ARHGAP5, ARHGAP31 and SRGAP2;

- the RHO GEFs ARHGEF5 and ARHGEF17;

- the RHO GAPs ARHGAP21 and ARHGAP35 (p190RhoGAP);

- the RAC/RHO GEFs DOCK5 and TRIO (ARHGEF23);

- and the multifunctional protein BCR with GEF, GAP and kinase activities located in different domains 54, 55.

As illustrated in Fig. 5A, activated RHO-ROCK and RAC-PAK pathways impact actin filament (F-actin) polymerization (via RAC - ARPC1B/2/3, p38 - HSP27) and stabilization (via ROCK - LIMK2), actin network assembly (via ROCK - MSN and PKC - MARCKS) as well as F-actin membrane attachment (via ROCK - ADD1, MSN and MARCKS). Besides these mechanisms affecting F-actin organization, the regulation of myosin contraction through the regulation of MLC2 phosphorylation by the opposing functions of MYLK (kinase) and MYPT1 (phosphatase) is another key feature of ROCK-controlled signaling. These findings have high biological relevance in the context of HGSC, as cancer cell attachment, migration and invasion are all actomyosin-governed processes.

Several proteins with LPA-regulated phosphosites further figure in multiple other signaling pathways, in particular ERK1/2 and PKCδ, suggesting that these protein kinases also play central roles in the LPA-controlled signal transduction network. This notion is supported by the observation that the targets of ERK and PKC include components of F-actin organization (such as F-actin-membrane crosslinking protein MARCKS) and myosin contraction (e.g., MYPT1). Furthermore, ERK and PKC play a prominent role in the direct or indirect regulation of both transcription and translation factors, which in turn are linked to cell proliferation, survival and stress response (Fig. 5B). ERK may also figure in the LPA-induced phosphorylation of ADAM17, potentially promoting pro-tumorigenic effects by proteolytic shedding (Fig. 5B).

### LPA-mediated regulation of MYPT1 and MLC2 phosphorylation

**Figure 6:**
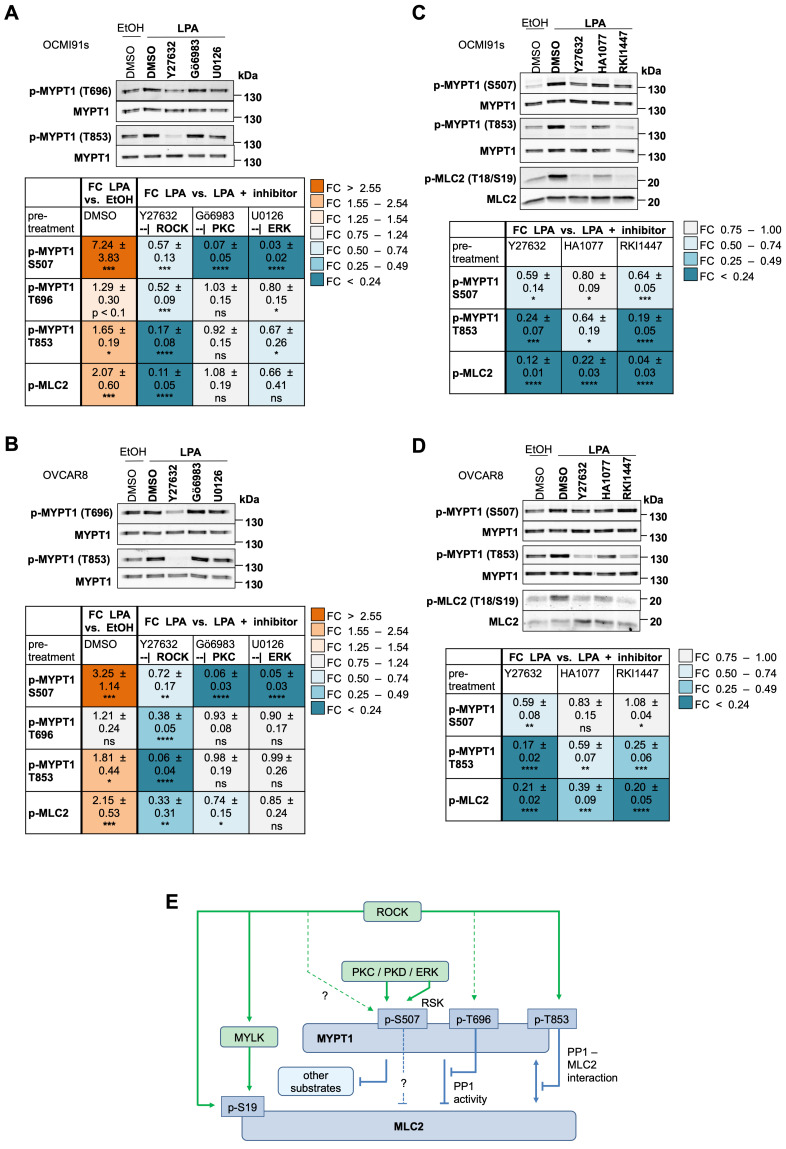
** Role of ROCK, PKC and ERK in LPA-induced MYPT1 phosphorylation. (A, B)** Effects of selective ROCK, PKC and ERK inhibitors on phosphorylation of MYPT1 at S507, T696 and T853 triggered by LPA. OCMI91s cells (panel A) and OVCAR8 cells (panel B) were treated with 10 µM of Y27632, Gö6983 or U0126 or solvent (DMSO) before stimulation with 5 µM LPA or solvent (EtOH) for 5 min. The data for S507 and p-MLC2 are from Fig. 4B and C and included here to allow for a direct comparison. **(C, D)** Comparison of the effects of different ROCK inhibitors (10 µM Y27632, 10 µM HA1077, 1 µM RKI1447) on LPA-induced phosphorylation of S507, T696 and T853 in OCMI91s cells (panel C) and OVCAR8 cells (panel D). Upper panels in A-D show representative immunoblots and the quantification of n = 4-5 biological replicates below. Colors reflect the fold change of phosphorylation relative to solvent control (left-most column) or relative to LPA (all other columns). Values represent the mean ± SD. Asterisks indicate p values determined by two-sided, paired t-test: **** p < 0.0001; *** p < 0.001; ** p < 0.01; * p < 0.05; ns: not significant. **(E)** Model summarizing the role of ROCK and other kinases in the LPA-induced phosphorylation of MYPT1 in OCMI91s cells. The indicated LPA-induced phosphorylation sites in MYPT1 and MLC2 (Figs. 5, 7A-D; Table S7) agree with published regulatory sites 62, 63, 66, 67, 104-107. ROCK-dependent induction of T696 phosphorylation by LPA is comparatively weak, as indicated by a dashed line. Phosphorylation of S507 of MYPT1 (panels A and C) is dependent on both PKC and ERK, and to a considerably lesser extent on ROCK (indicated by dashed line), consistent with the described role of RSK in S-507 phosphorylation 107. ?: unclear connection. As S507 has not been identified as ROCK target site, the observed effect may be indirect or due to off-target effects of Y27632. The indicated enhancement of PP1 - MLC2 interaction is based on published observations 62. MYPT1 targets other than MLC2, including EZH2 111, PLK1 108 and regulators of the Hippo pathway 109 have been described.

In view of the apparently prominent role of LPA-mediated signal transduction in regulating signaling to the actomyosin system (Fig. 5), we focused our functional studies on these aspects. According to our phosphoproteome data (Fig. 2E; Table S8) and immunoblotting analysis (Figs. 3A, 3B, 4B and 4C), MYPT1 is phosphorylated by LPA. In view of its pivotal role of in MLC2 regulation, cell adhesion, migration and cancer progression 65, 66, 68, we performed a detailed analysis of the regulation of MYPT1 phosphorylation in response to LPA. To this end, we analyzed the phosphorylation of the established ROCK target sites T696 and T853 62, 63, 66, 67 by immunoblotting, as the phosphoproteome data for these sites were inconclusive (Table S7: T853 not present; T696 not significantly regulated by LPA, but responsive to H2L with FC = 1.27 and p = 0.028). The immunoblotting data in Fig. 6A and B show that phosphorylation of T853 was significantly induced by LPA in both OCMI91s and OVCAR8 cells. Phosphorylation of T696 was also upregulated by 1.2- to 1.3-fold in both cell lines, but this increase remained below the statistical significance threshold (Fig. 6A and B), which is consistent with the phosphoproteomics data alluded to above.

While the ROCK inhibitor Y27632 strongly decreased phosphorylation of T853 in OCMI91s and OVCAR8 cells (83% and 94% reduction, respectively), its effect on T696 was comparatively low (48% and 62%, respectively), and even weaker on S507 (43% and 28%, respectively). In view of potential off-target effects of Y27632, we tested two other ROCK inhibitors, i.e., HA1077 and RKI1447 69, which confirmed the effect on both T853 and MLC2 phosphorylation observed with Y27632 (Fig. 6C and D). In contrast to Y27632, Gö6983 and U0126 hardly affected phosphorylation of MYPT1 at T696 and T853 and only weakly inhibited MLC2 phosphorylation (0-34% versus 67-89% for Y27632). Furthermore, the effect of HA1077 and RKI1447 on S507 phosphorylation were inconclusive, suggesting that off-target effects may be involved in the observed inhibition of S507 phosphorylation by Y27632.

These findings are graphically summarized in Fig. 6E. The data that ROCK signaling plays a pivotal role in LPA-mediated MLC2 regulation by inhibitory phosphorylation of MYPT1 (in particular of T853) as well as direct phosphorylation of MLC2. In contrast, the PKC/ERK-mediated phosphorylation of S507 in MYPT1 plays a minor role in MLC2 regulation.

### MYPT1 in LPA-mediated regulation of actomyosin dynamics

**Figure 7:**
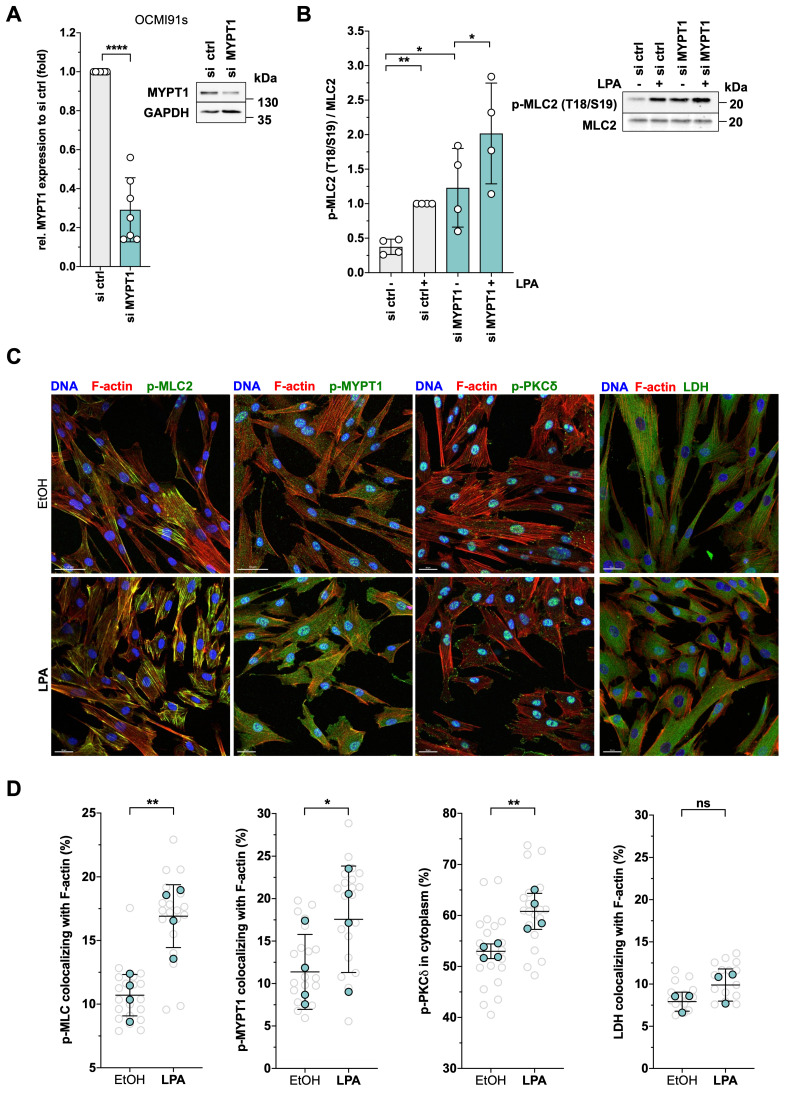
** Role of MYPT1, MLC2 and PKC in actomyosin dynamics. (A)** Role of MYPT1 in the LPA-induced MLC2 phosphorylation. OCMI91s cells were transfected either with siRNA control (si ctrl) or siRNA targeting *MYPT1* for 48 h and inhibition of MYPT1 expression was validated by immunoblotting (left panel: n = 7; insert shows representative immunoblot). **(B)** Quantification of p-MLC2 in LPA-treated cells based on n = 4 biological replicates. The plot shows the mean ± SD. Asterisks indicate p values determined by two-sided, paired t-test: ****p < 0.0001; ***p < 0.001; **p < 0.01; *p < 0.05; ns: not significant. **(C)** Representative pictures of immunofluorescence staining of nuclear DNA (blue), actin filaments (red) and phosphoproteins (green) after stimulation of OCMI91s cells with 5 µM LPA or solvent (EtOH) for 1 h. Staining of LDH was included to control for coincidental colocalization. Scale bar: 30 µm. **(D)** Quantification of the immunofluorescence staining in panel C. Shown is the mean (green dots) ± SD of n = 3-4 biological replicates with 5 random areas analyzed per replicate (grey dots).

siRNA-based loss-of-function experiments confirmed the functional relevance of MYPT1 in controlling MLC2 phosphorylation. MYPT1-siRNA increased the level of phosphorylated MLC2 in solvent-treated cells 3.2-fold and 2.0-fold in LPA-induced cells (Figs. 7A and B). The data suggest that suppression of the basal activity of MYPT1 in OCMI91s cells (by phosphorylation or siRNA) is required to achieve full activation of MLC2.

In agreement with the phosphorylation of MYPT1 and MLC2, staining of actin filaments confirmed a contracted phenotype of OCMI91s cells after LPA treatment (Fig. 7C, bottom row showing smaller contracted cells). Further in line with these findings, phosphorylated MLC2 was increased by LPA and relocated to actin filaments, in accordance with a contraction-promoting role (Fig. 7C, left; quantification in Fig. 7D). Moreover, LPA treatment also induced phosphorylated MYPT1, which also colocalized with F-actin (Fig. 7C and 7D). Furthermore, phosphorylated PKCδ relocated from the nucleus to cell edges (Fig. 7C and D), including actin-membrane junctions, consistent with a function in cytoskeletal dynamics.

Colocalization of these phosphoproteins with actin structures was validated by staining for lactate dehydrogenase (LDH) as a negative control, which showed ~8% colocalizing signals compared to ~15-20% for p-MLC2 and p-MYPT1. Furthermore, no significant difference between solvent and LPA treatment was observed for LDH (Fig. 7C and D). Moreover, quantification of total F-actin yielded very similar values for LPA-induced and solvent-treated cells (Fig. S6).

### Distinct roles for ROCK, PKC, ERK and MYPT1 in OC cell migration

**Figure 8:**
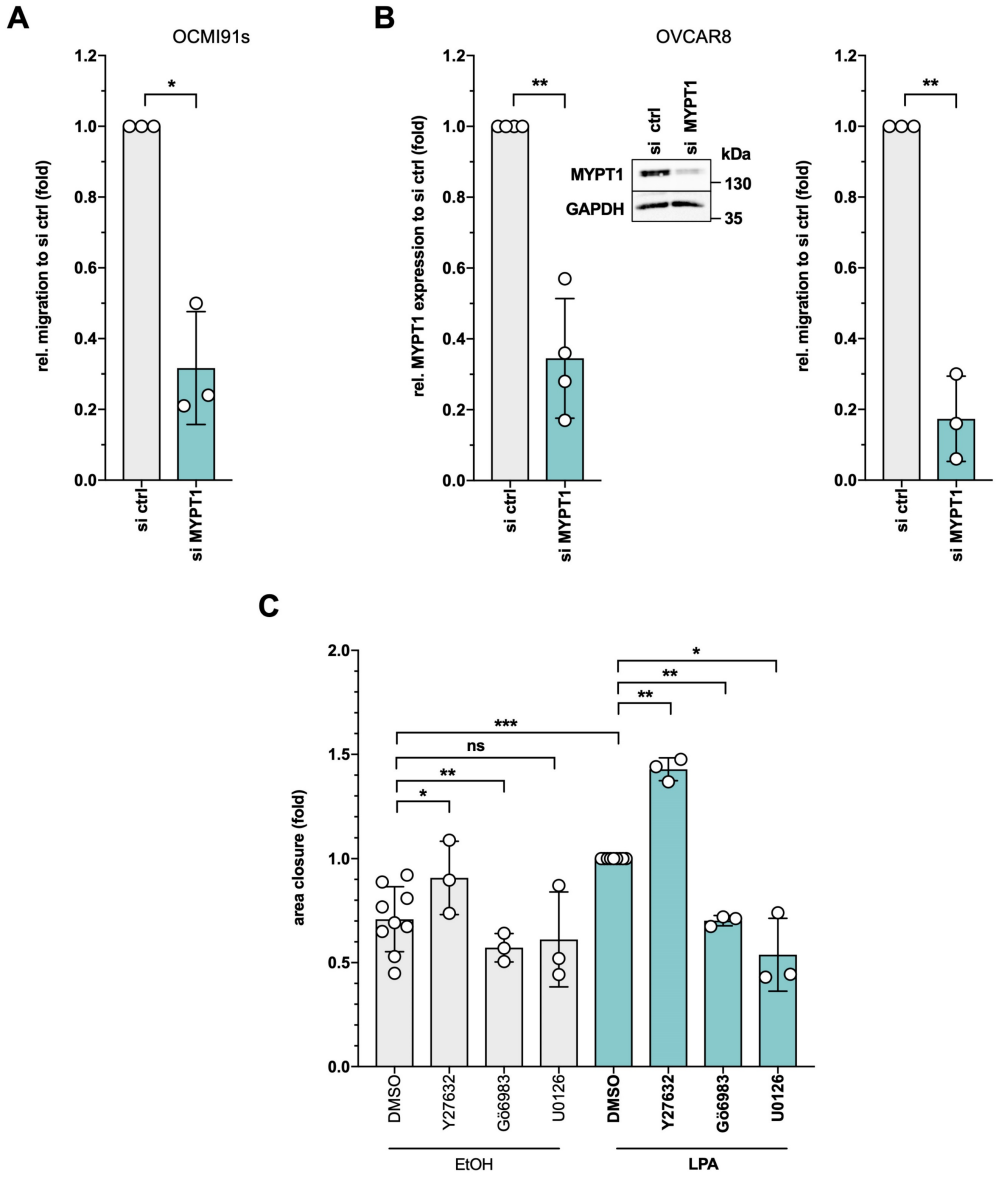
** Role of MYPT1 and upstream kinases in OC cell migration. (A)** Role of MYPT1 in the LPA-induced migration. OCMI91s cells were transfected either with siRNA control (si ctrl) or siRNA targeting *MYPT1* for 48 h (see Fig. 7A for siRNA validation), seeded into transwell inserts and allowed to migrate towards 5% FCS for 8 h (right panel). **(B)** OVCAR8 cells were treated as in panel A, except that migration was for 24 h towards 10% FCS. Representative pictures of migration assays are presented for both cell lines in Fig. S7. **(C)** Quantification of wound closure capacity of OCMI91s cells (Fig. S8) treated with Y27632, Gö6983, U0126 (all 10 µM) or solvent (DMSO) for 30 min before stimulation with 5 µM LPA or solvent (EtOH) for 8 h. All plots show the mean ±SD for n = 3 biological replicates. Asterisks indicate p values determined by two-sided, paired t-test: ***p < 0.001; **p < 0.01; *p < 0.05; ns: not significant.

Given the pivotal role of cytoskeletal dynamics in cell migration, we interrogated the function of MYPT1 in a transwell migration assay. As shown by siRNA interference experiments, MYPT1 plays a crucial role in LPA-induced migration of both OCMI91s cells (Figs. 8A and S7A; ~70% reduced migration) and OVCAR8 cells (Figs. 8B and S7B; ~80% reduced migration).

Due to their prominent role in the LPA signaling network and MYPT1 regulation, we additionally investigated the functional contribution of ROCK, PKC and ERK to LPA-triggered OC cell migration of OCMI91s cells in a wound closure assay (Fig. S8). As shown in Fig. 8C, the LPA-mediated induction of migration into the cleared area was counteracted by inhibition of either PKC (Gö6983) or ERK (U0126), while Y27632 significantly enhanced wound closure (Fig. 8C), impaired tail retraction (Fig. S9) and reduced cytoskeletal contraction (Fig. S10). Similar effects of ROCK inhibition have been reported for other experimental systems 70-73 and may relate to the observation that a requirement for ROCK depends on the mode of migration 74.

### Requirement for MYPT1 and DOCK7 in LPAR2-dependent entosis

**Figure 9:**
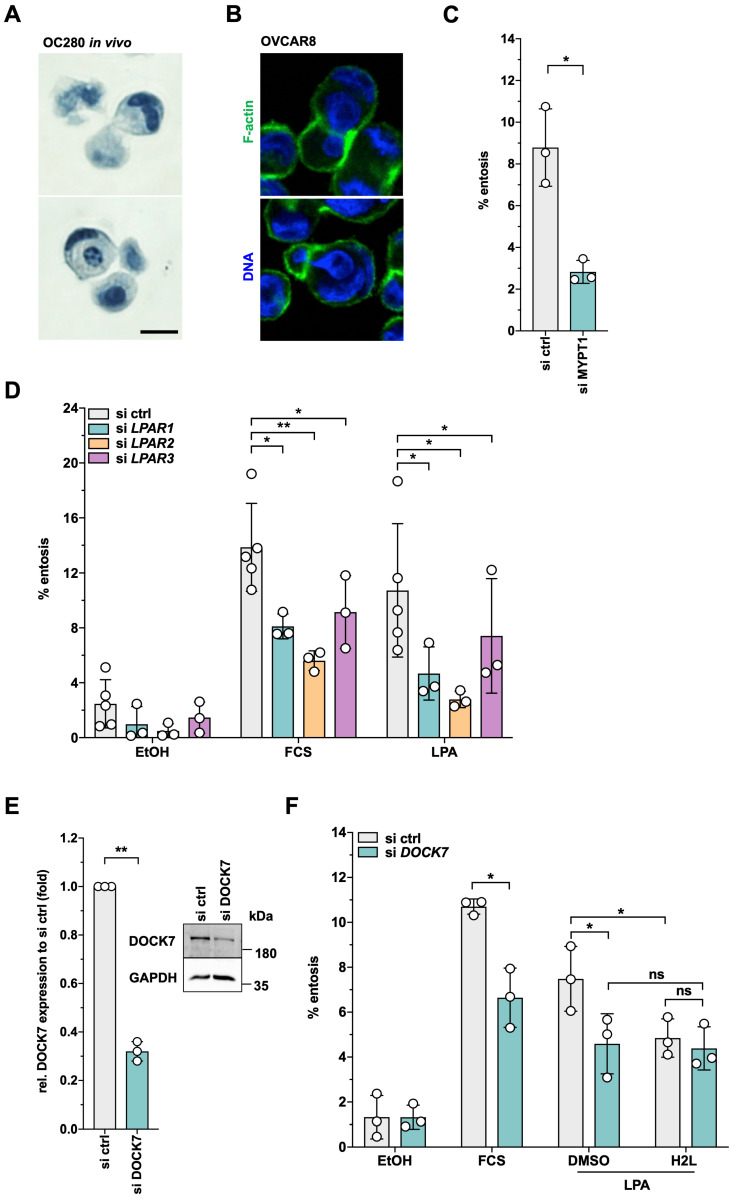
** Role of MYPT1 and a LPAR2-DOCK7 axis in LPA-triggered entosis. (A)** Detection of entosis in formalin-fixed spheroids from a HGSC patient (OC280) stained with hematoxylin. **(B)** Representative picture of entotic event in OVCAR8 cells after 4 h suspension culture in medium containing 15% FCS. **(C)** Crucial role for MYPT1 in LPA-triggered entosis. OVCAR8 cells were transfected either with siRNA control (si ctrl) or siRNA targeting *MYPT1* for 48 h (efficiency documented in Fig. 8B). To induce entosis, the cells were plated on ultra-low attachment plates for 4 h and stimulated with 5 µM LPA. **(D)** Essential roles for LPAR1 and LPAR2 in LPA-induced entosis. OVCAR8 cells were double-transfected either with siRNA control (si ctrl) or siRNA targeting *LPAR1, LPAR2* or* LPAR3* for 72 h (efficiencies documented in Fig. S11). To analyze entotic events, the cells were plated out into ultra-low attachment plate for 4 h and stimulated with 15% FCS, 5 µM LPA or solvent (EtOH). The entosis-promoting potential of serum has previously been reported to be due to its high content in LPA 41, which is confirmed by the data in this panel. **(E)** Validation of DOCK7 siRNA. OVCAR8 cells were transfected either with siRNA control (si ctrl) or siRNA targeting *DOCK7* for 72 h and the inhibition of expression was validated with Western Blot (see insert). **(F)** Essential role for DOCK7 in LPA-triggered entosis. OVCAR8 cells were transfected either with siRNA control (si ctrl) or siRNA targeting *DOCK7* for 72 h. To induce entosis, the cells were plated on ultra-low attachment plates for 4 h and stimulated with 5 µM LPA. Additionally, the cells were pretreated with 1 µM H2L or solvent (DMSO) before stimulation with 5 µM LPA (n = 3 biological replicates). Plots show the mean ± SD. Asterisks indicate p values determined by two-sided, paired t-test: **p < 0.01; *p < 0.05; ns: not significant.

Entosis is another actomyosin-dependent process relevant to tumor progression 45 and promoted by LPA 41, but has not yet been studied in HGSC. In our cohort of HGSC patients, entotic events were readily detectable in spheroids from HGSC patients (Fig. 9A) as well as in patient-derived HGSC cells cultured under non-adherent conditions and analyzed by time-lapse microscopy as described 41 (see Supplementary Video: entotic cell-in-cell invasion at the bottom left corner). Similar observations were made with the established HGSC cell line OVCAR8 under non-adherent conditions (Fig. 9B). Intriguingly, siRNA-mediated MYPT1 inhibition significantly decreased the fraction of entotic events in OVCAR8 cells (Fig. 9C), hinting at a central role of phosphorylation-controlled actomyosin dynamics in entosis.

A previous study of LPA-mediated entosis in breast cancer MCF10A cells revealed LPAR2 as an LPA receptor essential to the process 41. Our phosphoproteome analysis identified DOCK7 as one of the few proteins, whose LPA-induced phosphorylation was selectively H2L-sensitive (Fig. 2E). We therefore tested whether LPAR2 and the DOCK7 guanine nucleotide exchange factor (GEF) of RAC GTPases may be involved in LPA-mediated entosis in OVCAR8 cells. As shown by Fig. 9D, siRNA-mediated inhibition of either LPAR1, LPAR2 or LPAR3 expression (validation in Fig. S11) significantly inhibited entosis, but the strongest inhibitory effect was observed with LPAR2 siRNA (FC ~3). Likewise, inhibition of DOCK7 expression (Fig. 9E) resulted in a significantly diminished fraction of entotic events (Fig. 9F). H2L had a very similar effect without any additive effect when combined with siRNA, validating both the siRNA and the inhibitor and confirming DOCK7 as an essential component of LPAR2-induced signal transduction.

**Figure 10:**
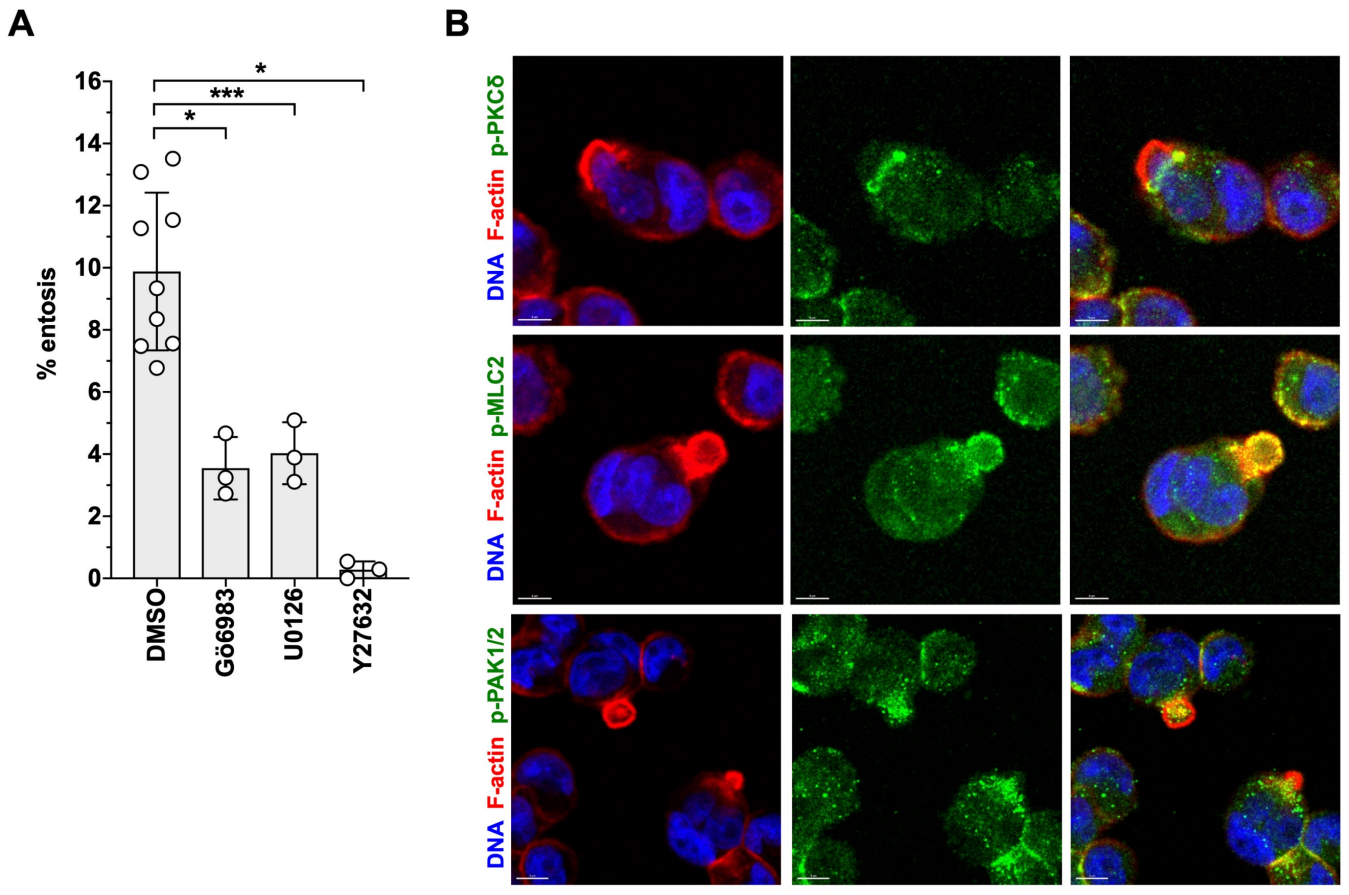
** Role of ROCK, PKC and ERK signaling in migration and entosis. (A)** Effect of Y27632, Gö6983, U0126 on entosis of OVCAR8 cells. Plots in A and B show the mean ± SD. Asterisks indicate p values determined by two-sided, paired t-test: *** p < 0.001; * p < 0.05. **(B)** Localization of phosphorylated forms of MLC2, PKCδ and PAK1/2 in entotic events. Representative image of immunofluorescence staining of nuclear DNA (blue), actin filaments (red) and the indicated phosphoproteins (green). Scale bar: 8 µm.

Both, Gö6983 and U0126 had similar inhibitory effects on LPA-triggered entosis. As shown in Fig. 10A, pharmacological inhibition of PKC or ERK significantly reduced entotic events by more than 60%, concomitant with the localization of phosphorylated PKCδ in the mechanical ring 40 of entotic OVCAR8 cells (Fig. 10B). In agreement with the known instrumental role of RHO-ROCK in entosis 43, 44, 75, the ROCK inhibitor Y27632 blocked LPA-induced entosis by > 95% (Fig. 10A) and p-MLC2 accumulated in the mechanical ring of LPA-induced cells (Fig. 10B). Finally, consistent with the LPA-induced phosphorylation of the RAC-GEF DOCK7 (Figs. 2E and 5A), phosphorylated PAK2 (a RAC1 substrate) was also detected in entotic events (Fig. 10B).

## Discussion

LPA is found at high concentrations in the HGSC microenvironment and is associated with metastatic progression and a shorter time to relapse 12, 16, 17. Even though LPA is known to trigger metastasis-promoting biological processes via multiple signal transduction pathways 14, 24-35, 40, the LPA-regulated signaling network in HGSC cells remained to be analyzed by unbiased approaches. In the present study, we addressed this issue by mass spectrometry-based phosphoproteomics validated by loss-of-function approaches in conjunction with RNA-sequencing, biochemical assays and cell imaging. These studies provided the basis for drafting the LPA-regulated signal transduction network and linking specific pathways to cancer-relevant biological processes, such as actomyosin dynamics, cell migration and entosis.

### A draft of the LPA-regulated signal transduction network

Based on our phosphoproteomics data, we defined 721 proteins with LPA-regulated phosphosites in patient-derived HGSC cells (OCMI91s; Fig. 2B). A previous study 47 identified 120 proteins with LPA-regulated phosphorylation sites in A498 kidney carcinoma cells, 43 of which were also found in OCMI91s cells, while 664 of the sites detected in OCMIs cells were not observed in the A498 phosphoproteome (Fig. S12). The latter include key phosphoproteins, such as ARHGAP35, PKC, PPP1R12A (MYPT1) and TRIO (Figs. 4A and 5). We attribute this difference to the considerably greater depth of the LPA-regulated phosphoproteome in our study, as well as potential specificity of features to the cancer entity studied.

By integrating the LPA-regulated phosphoproteins with literature information we have established the putative LPA-regulated signal transduction network and discuss speculative aspects of it in the following.

First, the model incorporates components which were not detected in the phosphoproteome but integrated based on knowledge from the literature and partly validated by immunoblotting, as for MLC2 and AKT (Fig. 3A and B; Table S7). We attribute the omission of these phosphoproteins from the phosphoproteome data set, respectively the absence of statistically significant changes in phosphorylation, to technical reasons such as the non-exhaustive nature of the phosphoproteomic screen and the frequently high variance of measurements combined with a limited number of replicates.

Second, due to the sheer number of phosphosites identified in combination with the lack of available corresponding immunoreagents, we were only able to verify a limited number of candidate sites and proteins from the phosphoproteome data. As all validation was successful and corroborated in part by analysis of further downstream effects (Fig. 3C, D), we consider the data from the phosphoproteomics analysis reliable.

Third, proteins may contain multiple phosphorylation sites regulated independently in response to LPA (Fig. 2B). Whether the integration of those signals results in net positive or negative regulatory effects often remains unclear. For phosphosites identified in several key proteins in our model, however, the regulatory impact has been literature-reported and is consistent with the proposed pathway (e.g., PKCδ).

Fourth, the functional connections in Fig. 5 are supported by published observations, some of which were experimentally verified, such as the effect on actomyosin dynamics and entosis. Other suggested functional links appear, however, worthy of further investigation in future, given the extent of literature-conformity and successful validation in the performed follow-up. Comparison of the phosphoproteomes of OCMI91s and OVCAR8 cells also suggests LPA-induced phosphorylation of many of the key regulators to be model-independent (Fig. 4A). Taken together, these considerations render the network delineated in Fig. 5 a suitable basis of the continued exploration of LPA-regulated signaling in HGSC cells. We subsequently discuss salient features of this network as well as the putative role of central components.

### Roles of GPCR-linked pathways by LPA in HGSC cells

As typical for GPCRs, LPARs couple to several Gα proteins (G_s_, G_i/o_, G_q/11_, G_12/13_), thereby targeting multiple effector proteins, including phospholipase C, phosphatidylinositol 3-kinase (PI_3_K), adenylate cyclase and RHO 15, 59. Consistent with this general model, we found LPA to stimulate a spectrum of downstream signal cascades. One of these is the release of intracellular of Ca^2+^ in HGSC cells (Fig. 3C), presumably as a consequence of phospholipase C activation by G_q/11_ and/or G_i/o_. This notion is supported by the observed LPA-induced increase in phosphorylation of the phospholipase C isoform PLCB3 (Fig. 5; Table S8). Further downstream mediators of Ca^2+^ release remain currently unclear, as PKCδ, the only PKC isoform significantly affected by LPA-regulated phosphorylation, is a Ca^2+^-independent enzyme 76, and calmodulin-dependent kinases (CAMKs) were not found among the LPA-regulated proteins (Tables S7-S9). It is likely that the second product of PLCB3, diacyl glycerol (DAG), stimulates PKCδ, which may lead to its autophosphorylation 77 (Fig. 5; Table S8).

LPA also increased the level of cAMP in HGSC cells (Fig. 3D), which in concert with phosphorylated A-kinase anchoring protein 13 (AKAP13) is likely to activate PKA signaling. LPA also stimulated AKT phosphorylation (Fig. 3A, B), consistent with the activation of PI_3_K via G_i/o_ 59. The same G-protein is also able to activate RAS-MAPK signaling, consistent with the observed phosphorylation of multiple components of MAPK signaling (see paragraph below). Finally, we observed increased activation of the ROCK pathway (Fig. 5; Tables 2 and 3), which can be activated via G_i/o_, G_q/11_ and/or G_12/13_ 59, and is discussed in detail further below.

Our data indicate that pathways controlled by ROCK, MAPKs and PKC are central to LPA-triggered signal transduction, as suggested by several lines of evidence: (i) a central function of PKC and MAPK in transcriptional signaling is predicted by upstream pathway analysis (Table S6). In agreement with this prediction, we found LPA-upregulated phosphorylation sites on several kinases acting upstream of MAPK-regulated transcription factors (Table 2), i.e., MAPK1 (ERK2), MAPK3 (ERK1), MAP2K1/2 (MKK1/2), MAP2K7 (JNKK2), MAP3K4 (MEKK4), MAP4K4 (MEKKK4) and MAPK14 (p38). Moreover, JUN and/or JUND were phosphorylated on S73 (Table S8), known as target site of JNKK 78, 79. The functional relevance of these observations was confirmed by reduced expression of two LPA target genes analyzed in detail, i.e., *IL6* and *THBS1,* through inhibition of MAPKs, PKC and its target PKD 80 (Fig. 1C and D).

*THBS1* expression was also inhibited by the ROCK inhibitor Y27632 (Fig. 1), which is likely due to activation of the serum-response factor SRF 81, 82. SRF is a transcription factor forming a complex with the MRTFA (MAL) coactivator to target genes regulating the cytoskeleton and cell migration. MAL is under control of RHO-induced changes in actin organization: it is sequestered by globular actin in the cytoplasm, preventing its interaction with SRF in the nucleus, while formation of F-actin releases MAL, enabling its function as SRF coactivator. The *THBS1* promoter has been shown to harbor a SRF binding site 83, consistent with a role of ROCK in its LPA-induced transcription.

### Regulation of F-actin dynamics by LPA

Pathways governed by RHO GTPases, and thus the regulation of different steps of F-actin organization via their downstream effectors, are among the most frequent targets of LPA-triggered signaling (Fig. 5). Modulation of both RHO and RAC pathways through LPA is suggested by the observed changes in the phosphorylation status of their respective effector kinases, LIMK2 and PAK2 (Tables 2 and S7). Phosphorylation of PAK2 (S2, S19) has also been described in other experimental systems, but its functional significance remains unclear 84. LIMK2 is phosphorylated by ROCK at T505 to stimulate its kinase activity towards cofilins 85, 86. We found the identical site phosphorylated in response to LPA (Fig. 3A and B). Phosphorylation of cofilins in turn inhibits their F-actin-severing activity and thus stabilizes the cytoskeleton to regulate actin-dependent biological processes 87. Another LPA-regulated phosphoprotein relevant in this context is the small heat-shock protein HSP27 (HSPB1), which directly interacts with actin and stabilizes and protects microfilaments when organized in phosphorylated oligomers 88. Our phosphoproteomic analysis identified S15, S82 and S86 as LPA-induced phosphosites, and previous studies reported that phosphorylation of at least two of these sites are required for F-actin rearrangement 89, 90. HSP27 is a substrate for p38 and PKD 89, 91, 92, which are both activated by LPA (Fig. 3) and therefore likely to contribute to HSP27 phosphorylation.

Other LPA-regulated phosphoproteins promote actin polymerization, such as ARPC1B (Tables 2 and S8), a component of the ARP2/3 multiprotein complex that mediates nucleation of actin polymerization and branching, thereby providing the force for cell motility 93. The ARP2/3 complex is activated by the RAC-regulated WAVE complex 94, constituents of which include at least 3 proteins with LPA-induced phosphosites, i.e., ABI1, ABI2 and WASF2 (Tables 2 and S8). ARP2/3 can also be activated via the WASP complex, which in turn is regulated by CDC42, another member of the RHO GTPase family. Our data indicate that the CDC42 effector protein CDC42EP1 is an LPA-upregulated phosphoprotein with functions in cell shape regulation 95, potentially providing a further link between LPA-signaling and F-actin organization. F-actin crosslinking is further promoted by MARCKS 96, which is also among the proteins with upregulated phosphosites in our screen (Tables 2 and S8). MARCKS is one of the most abundant substrates of PKC, and is also phosphorylated by the Ca2+-independent PKCδ isoform 97. One of the phosphorylation sites has been mapped to S170 97, which we find upregulated by LPA (Table S8), suggesting a LPA - PKCδ - MARCKS pathway.

Actin-dependent biological processes also depend on the connection between cytoskeleton and plasma membrane. At least two proteins with LPA-induced phosphosites figure in this process. These are moesin (MSN), an ezrin-radixin-moesin family protein, and adducin 1 (ADD1), which both promote F-actin-membrane linkage (Tables 2 and S8) and are regulated by ROCK 98-100 as well as PKA and PKC 101. As the phosphorylation sites identified in our study differ from the published sites on MSN and ADD1, it remains to be investigated if the proposed functional links extend to HGSC cells.

Taken together, our observations indicate that LPA affects the actin networks by targeting a range of mechanisms and signaling events with clear implications for actin-dependent biological processes.

A conspicuous feature of the LPA-regulated signaling network is the large number of direct regulators of RHO and RAC 102 including 13 GEFs and GAPs (Fig. 5; Tables 2 and S8). Their regulation by phosphorylation has been reported in the Phosphosite database 84, but in most cases the functional consequences of LPA-induced phosphorylation are unknown and need further investigation. Several of the RHO/RAC regulators harbor multiple LPA-regulated phosphorylation sites, indicating prominent role in the LPA-mediated regulation of RHO/RAC GTPases. These include the ARHGAP5, TRIO and ARHGEF17 with three LPA-regulated phosphosites, as well as DOCK7 with six regulated phosphosites (Tables S8 and S9). The latter is of particular interest in view of its role in entosis, as discussed further below.

### LPA-mediated regulation of myosin

Cell shape, the generation of contractile forces as well as motility are mediated by the myosin II component of actomyosin 103. Myosin contraction is regulated by the phosphorylation status of its light chain (MLC, MYL) through opposing mechanisms, i.e., activating phosphorylation by myosin light chain kinase (MYLK in non-muscle cells), and inhibitory dephosphorylation by PP1. The latter in turn is regulated by ROCK-mediated phosphorylation of its regulatory subunit MYPT1 (PPP1R12A), which reduces its catalytic activity and/or association with myosin 60, 61, 64.

LPA-treated HGSC cells exhibit a contracted phenotype (Fig. 7A), suggesting cytoskeleton-associated myosin as a target of LPA-triggered signaling. Our data (schematic summary in Fig. 6E) suggest that ROCK signaling is instrumental in LPA-mediated MLC2 regulation mainly via two pathways: (i) downregulation of MYPT1 activity by phosphorylation of MYPT1 at T853 and (ii) direct phosphorylation of MLC2, which is a known substrate of ROCK 104-106. In contrast, phosphorylation of MLC2 was only weakly, if at all, affected by PKC (Gö6983) and ERK (U0126) inhibition. These findings also suggest that phosphorylation of S507 in MYPT1 by PKCδ/ERK is less relevant with respect to LPA-mediated MLC2 regulation than ROCK signaling. These findings differ from those of Samson et al. who reported that RSK-induced phosphorylation of S507 stimulates T853 phosphorylation, inhibits PP1-myosin interaction and thus increases MLC2 phosphorylation 107. It is possible that differences in the experimental systems account for this discrepancy, i.e., unmodified HGSC cells in the present study and overexpression systems using 293T in the study by Samson and colleagues 107.

However, as phosphorylation of S507 is strongly induced by LPA (3-to 7-fold), which is completely abrogated by Gö6983 and U0126 (Fig. 6A and B), a role for a PKCδ/ERK -| MYPT1 signaling in pathways other than MLC2 regulation is likely. This hypothesis is compatible with the identification of MYPT1 targets other than MLC2 108-111 and is discussed in more detail below. S507 phosphorylation was also inhibited by Y27632, albeit with a considerably lower effect compared to p-MLC2 (Fig. 4B and C). As this phosphorylation site has not been identified as a ROCK target in previous studies, the observed inhibition by Y27632 may reflect an indirect signaling mechanism. Finally, the effect of the ROCK inhibitors HA1077 and RKI1447 on S507 phosphorylation was weak or not significant (Fig. 6C and D), while their effect on T853 phosphorylation was comparable to Y27632, suggesting that inhibition by Y27632 may be due to off-target effect(s).

MLC2 is also phosphorylated by the Ca^2+^/calmodulin-regulated kinase MYLK 112. We observed intracellular release of Ca^2+^ in LPA-stimulated HGSC cells, potentially further linking LPA signaling to MLC2. Furthermore, MLC2 is a target of DAPK1 113. DAPK1 is phosphorylated in response to LPA (Tables 2 and S7; Fig. 5), and may thus play a role in LPA-regulated myosin dynamics. Finally, MYLK is also subject to inhibitory phosphorylation by PAK2 114. We detected LPA-mediated downregulation of phosphorylation at T335. Even though the function of this particular phosphorylation site remains unknown, our finding provides a potential link of MYLK to RAC - PAK signaling.

### Regulation of cell migration by LPA

Cancer cell migration is an actomyosin-dependent process, consistent with the widespread effects of LPA on RHO GTPase signaling, actin and MLC2 (Fig. 5) with a major role for LPAR1 (Figs. 2 and 3). Intriguingly, Y27632 significantly enhanced migration in a wound closure assay (Fig. 8C). This is consistent with previously published observations showing that ROCK and/or RHOA inhibition promotes the migration of tumor cells 71, 72 and fibroblasts 73, which may be due to a migration-mode-dependent requirement for ROCK 74. Furthermore, ROCK inhibition has been reported to induce an elongated morphology in conjunction with impaired tail retraction 70, both of which is also observed in LPA-treated OCMI91s cells (Figs. S9 and S10).

These findings suggest that ROCK is essential for MLC2 phosphorylation, either directly or indirectly by inhibiting MYPT1 (Fig. 6C, D), but is dispensable for migration, which is even enhanced by ROCK inhibition (Fig. 8C). Conversely, inhibition of MYPT1 expression resulted in enhanced MLC2 phosphorylation (Fig. 7B) and suppressed cell migration (Fig. 8A and B). The latter may result from MLC2 "hyperactivity" causing a contracted state impeding migration. This hypothesis is supported by the observation that excessive tension induced by high myosin II activity reduces membrane protrusion and migration velocity 115. However, functions of MYPT1 other than MLC2 regulation may also be involved 108-111. Of particular interest in this context may be the reported regulation of the Hippo pathway by MYPT1 116, as the Hippo-regulated transcription factor TAZ (WWTR1) is subject to LPA-regulated dephosphorylation (Table S9), and Hippo signaling has been implicated in cytoskeletal organization 117, cancer cell migration 116 as well as OC progression 118. Finally, our data indicate that OCMI91s cell migration is dependent on PKC and ERK (Fig. 8C), as is the LPA-induced phosphorylation of S507 in MYPT1 (Fig. 4B). At present, the functional links between MYPT1, S507 phosphorylation, PKC/ERK and their impact on signaling pathways regulating cell migration are unknown. However, our data provide the basis for unraveling the complexity of these interactions in future studies.

### Promotion of entosis by LPA

We also for the first time report the promotion of entosis by LPA in HGSC cells, consistent with the detection of entotic events in tumor cells spheroids from patients (Fig. 8A and B). RHOA/ROCK-controlled pathways and actomyosin contractility of the invading cell are the known driving force for the initiation of entosis 45, 46, which is in agreement with our observation that entosis of HGSC cells is dependent on both MYPT1 and ROCK (Fig. 9A and B). Thus, in contrast to ROCK, which is dispensable for migration, MYPT1 is essential for both migration and entosis. Whether the dependency on MYPT1 is linked to MLC2 regulation or other pathways (as discussed above for migration) remains to be investigated. Maximal LPA-induced entosis also requires PKC and ERK signaling (Fig. 10A). The role of these signaling pathways in entosis has not been reported as of yet, and thus their precise function in this context remains elusive. Whether PKC/ERK-mediated phosphorylation of S507 in MYPT1 plays a role entosis may be an intriguing question to be addressed in future studies.

We also identified a pathway required for LPA-driven entosis that is mediated by LPAR2 (Fig. 8C). Intriguingly, one of the few H2L-sensitive LPA-upregulated phosphosites is S439 of DOCK7 (Fig. 2E; Table S8), and the siRNA-mediated inhibition of DOCK7 expression significantly diminished LPA-induced entosis (Fig. 8D-E). A role for DOCK7 in migration has been described in neuroblasts, Schwann cells and U-87MG glioblastoma cells 119-121, but DOCK7 has not been previously linked to entosis.

DOCK7 is a RAC-GEF with the potential to regulate F-actin organization (Fig. 5A), consistent with previous publications reporting DOCK7-mediated RAC1/CDC42 - PAK1 activation in the DNA replication stress response 122, and the promotion of glioblastoma cell invasion by DOCK7 via RAC activation 123. These findings identify the LPAR2 → DOCK7 axis as a novel signaling mechanism regulating actomyosin dynamics to be of similar relevance in the initiation of entosis as LPAR1. As to how phosphorylation of S439 affects the function of DOCK7 in this context remains to be investigated, since the modified residue according to the Uniprot database 124 localizes to a protein domain of unknown function.

Entosis provides a potential survival advantage for the outer cell under unfavorable conditions, such as starvation or exposure to anti-cancer drugs 44-46. Intriguingly, entosis appears to have prognostic value in a majority of cancers, including head and neck, lung, pancreatic and breast carcinomas, where the frequency of entotic figures is associated with malignancy and a poor prognosis 45. Studies on the clinical significance of entosis in breast carcinoma have yielded seemingly contradictory results, which may result from divergent roles of entosis in different breast cancer subtypes 125, 126. Even though the clinical significance of entosis in HGSC is unclear, several lines of evidence hint at a tumor-promoting role. LPA is, for example, a potent inducer of entosis (Fig. 8A-C), and LPA levels are associated with a short relapse-free survival 12. Furthermore, both DOCK7 and MYPT1 are regulated by LPA (Figs. 2E and 3A) and play essential roles in LPA-induced entosis (Figs. 8E and 9A). These observations may be clinically significant in view of the reported overexpression of DOCK7 in OC 122 and the inverse association of *MYPT1* expression with HGSC survival (Fig. S13).

### Translational perspectives

RHO-GTPase-regulated actomyosin dynamics is a major driver of metastasis-associated processes, and therefore considered a drug target with the potential to improve cancer treatment 127, 128. Clinical evaluation of ROCK inhibitors, however, has been unsuccessful so far 129. It is likely that the development of ROCK-targeting drugs is hampered by a strong dependency on the experimental system and the microenvironment 130. Furthermore, ROCK inhibition may have undesired effects, as suggested by a described increase in monocyte crawling 70 and our own data demonstrating an Y27632-induced acceleration of wound closure *in vitro* (Fig. 7C). These observations suggest that the identification of alternative target molecules is required for developing new drug candidates interfering with actomyosin dynamics. This view is supported by a recent publication identifying TM9SF4 as an F-actin disassembly factor that promotes HGSC cell motility and metastasis and is required for metastatic growth in a mouse model 131.

Our data suggest MYPT1 to represent a further attractive novel drug target for HGSC therapy. Targeting MYPT1 inhibits tumor cell migration (Fig. 7D-E), which is required for metastasis formation, and blocks entosis (Fig. 9A). Even though the association of entosis with progression of HGSC remains to be investigated, entosis may contribute to cancer cell survival under stress conditions (such as chemotherapy) 45, 46 as well as to their escape from immune surveillance 132. Furthermore, MYPT1 phosphorylation is induced by LPA in both OCMI91s and OVCAR8 cells with different LPAR expression profile (Fig. S1), which is also characteristic of HGSC patients 12, suggesting that targeting MYPT1 may be preferable to LPAR-selective antagonists.

MYPT1 is a regulatory subunit of PP1, the latter previously proposed as a drug target in cancer 133, 134. PP1, however, appears to regulate more than 200 proteins 135, suggesting that the inhibition of its catalytic activity may have severe side effects. As the substrate specificity of PP1 is regulated by its subunit composition, the selective targeting of MYPT1 may provide a high degree of selectivity. In this context, proteolysis targeting chimeras (PROTACs) may be of particular interest, since these compounds work efficiently with proteins lacking enzymatic activity, which as a result previously were considered "undruggable", and have recently been shown to function in animal models 136, 137.

Taken together, these considerations suggest that the identification of MYPT1 as a pivotal mediator of LPA-triggered processes associated with HGSC progression paves the way to the development of novel therapeutic options.

## Supplementary Material

Supplementary figures and tables, video.

## Figures and Tables

**Table 1:** Pharmacological inhibitors used in the present study.

Name	Concentration	Targets	Gene names of targets
Ro6842262	1 µM	LPAR1	LPAR1
H2L5186303	1 µM	LPAR2	LPAR2
Y27632	10 µM	ROCK1/2	ROCK1, ROCK2
HA1077	10 µM	ROCK1/2	ROCK1, ROCK2
RKI1447	1 µM	ROCK1/2	ROCK1, ROCK2
Gö6983	10 µM	PKCα, PKCβ, PKCγ, PKCδ, PKCζ	PRKCA, PRKCB, PRKCG, PRKCD, PRKCE
BIRB796 (Doramapimod)	10 µM	p38MAPK	MAPK14
U0126	10 µM	MEK1/2 (upstream kinase of ERK1/2)	MEK1, MEK2
CRT0066101	2.5 µM	PKD1/2/3	PRKD1, PRKD2, PRKD3

**Table 2:** ** Proteins with phosphosites upregulated by LPA**. Details in Fig. 2 and Table S8 (p < 0.1; FC > 1.2; LPA > LPA+Ro or H2L).

**Protein kinases** Kinome: n = 25	CDK7 DAPK1 DDR2 EPHA2 MAP2K1/2(MKK1/2) MAP2K7(JNKK2) MAP3K4 = MEKK4 MAP4K4(MEKKK4) MAPK1(ERK2) MAPK3(ERK1) MAPK14(p38) MARK2 MARK3 NEK9 OXSR1 PAK2 PRKAA1 PRKAR2A PRKCD(PKCδ) PRKD1(PKD1) RIPK2 STK10 STK38 TRIO WNK4
**Transcription factors** TF Database: n = 17	ARHGAP35 ARID5B BBX CHAMP1 CHD1 CHD2 GABPA GTF2I MEF2D NCOR2 NFIC NFIX SPEN STAT3 TEAD3 TMF1 YBX3
**RHO GTPase signaling** Reactome Pathways: n = 57	ABI1 ABI2 AKAP13 ANKLE2 ARHGAP5 ARHGAP21 ARHGAP31 ARHGAP35 ARHGEF2 ARHGEF5 ARHGEF10 ARHGEF17 ARPC1B BCR CAVIN1 CCDC88A CDC42EP4 CTTN DNMBP DOCK5 DOCK7 EFHD2 EMD EPHA2 ERBIN GJA1 LMNB1 LRRC41 MAPK1 MAPK3 MAPK14 MAPRE1 MYO9B NUP107 PAK2 PEAK1 PLIN3 PPP1R12A PRKCD PTPN13 RANBP2 RHPN2 SCRIB SLC4A7 SOS1 SPEN SRGAP2 STK10 STK38 TJP2 TOR1AIP1 TRIO UHRF1BP1L VAMP3 VIM WASF2 WASL
**Cytoskeletal organization** GO biological process complete: n = 78	ABI1 ABI2 ADD1 ANTXR1 ARHGAP35 ARHGEF10 ARHGEF17 ARHGEF2 ARHGEF5 ARPC1B BCR CAMSAP2 CARMIL1 CCDC6 CCDC88A CTTN CXADR DLG1 DOCK7 DPYSL3 EML3 EPB41 EPB41L5 EPS8 ERBIN EZR FAM83H FHOD1 FLNC GAS2L1 GJA1 KRT18 LIMA1 LIMD1 LMNA LMNB1 LMNB2 MACF1 MAP1A MAP1B MAP7D1 MAPRE1 MARCKS MARCKSL1 MARK2 MARK3 MICAL1 MSN NAV1 NEBL NF1 NUMA1 PALM PARD3 PAWR PCM1 PDE4DIP PDLIM7 PHACTR4 PHLDB2 PKP2 PPP1R12A RBM14 SHC1 SHROOM2 SIPA1 SIPA1L1 SLAIN2 SLC9A3R1 SPTBN1 SRGAP2 SSH3 STMN1 TJP1 VIM WASF2 WASL ZYX
**Migration and metastasis** IPA: n = 100	ADAM17 ADAR ADD1 AFAP1 AHNAK ARHGAP21 ARHGAP31 ARHGAP35 ARHGAP5 ARHGEF2 ARID5B ASAP1 BAG3 BCL10 BCR CCDC88A CD40 CDK7 CHD1 CTNND1 CTTN CXADR DAB2 DAPK1 DDR2 DDX3X DOCK5 DOK1 DPYSL3 EPB41 EPB41L5 EPHA2 EPS8 EZR FHOD1 FLNC GAB2 GBF1 GIPC1 GJA1 HDAC4 HDGF HECTD1 HMGN1 HSPB1 IGF2R IL1R1 IRS2 ITGAV KHDRBS1 KRT18 LIMA1 LMNA LMNB1 LMNB2 MAP1B MAP2K2 MAP4K4 MAPK1 MAPK14 MAPK3 MAPRE1 MARCKS MARCKSL1 MARK2 MBP MSN MTDH MYO9B NAV1 NDRG1 NF1 NFIC NFIX PAK2 PDCD4 PDE4DIP PEAK1 PHACTR4 PKP2 PLA2G4A PLCB3 PRKCD PRKD1 PTPN14 PTPRA RABGEF1 RAPGEF2 RHBDF2 RIPK2 RTN4 SHC1 SLC4A2 SLC9A3R1 SPAG9 STAT3 STMN1 TOP2B TP53BP2 TPR

**Table 3:** ** Proteins with phosphosites downregulated by LPA**. Details in Fig. 2 and Table S9 (p < 0.1; FC > 1.2; LPA < LPA+Ro or H2L).

**Protein kinases** Kinome: n = 19	ABL1 CSNK1D DAPK3 EPHA2 MAP4K4 MAPKAPK5 MINK1 MYLK PAK2 PIK3R4 PRKCD PTK2 RAF1 ROR2 RPS6KA3 SRPK2 STK10 STK39 TRIO
**Transcription factors** TF Database 55: n = 19	ANKZF1 CHAMP1 CHD1 CIC DNAJC2 ELK3 FOSL2 GATAD2B HSF1 MEF2D MEIS1 NCOA2 NCOR2 NFIC SAFB SMARCC2 WIZ YBX1 ZNF687
**RHO GTPase signaling** Reactome Pathways: n = 71	AABI1 ABI2 ABL1 ADD3 AKAP12 AKAP13 ARHGAP12 ARHGAP17 ARHGAP21 ARHGAP5 ARHGEF17 ARHGEF40 ARHGEF5 ARHGEF7 BAIAP2 BCR CAV1 CCDC88A CDC42EP4 CLASP1 CLASP2 CLIP1 CTTN DLG5 DNMBP DOCK7 DVL3 EMD EPHA2 ERBIN FARP1 FNBP1L GIT2 HNRNPC HSP90AA1 ITSN2 KLC2 KLC2 MYH10 MYH9 MYLK MYO9B NCOA2 PAK2 PCDH7 PEAK1 PGRMC2 PIK3R4 PLXNA1 PRAG1 PRKCD PTK2 PTPN13 RALGAPA1 RASAL2 RBBP6 SCFD1 SH3PXD2A SH3RF1 SLC4A7 SOWAHC SPTAN1 SPTBN1 SRRM1 STK10 TJP2 TOR1AIP1 TRIO TRIP10 UHRF1BP1L VIM
**Cytoskeletal organization** GO biological process complete: n = 87	ABI1 ABI2 ABL1 ADD1 ADD3 ADRA2A AKAP11 ALDOA ARHGAP12 ARHGAP17 ARHGEF17 ARHGEF5 ATRX BAIAP2 BCR CAP1 CCDC88A CETN2 CLASP1 CLASP2 CLIP1 CSNK1D CTTN DOCK7 DPYSL2 DPYSL3 DSTN EHD2 ENAH EPB41L1 EPB41L2 ERBIN FAM83H FARP1 FLNB GAPDH INF2 KANK1 LIMA1 LMOD1 LSM14A MAP1A MAP1B MAP1S MAP4 MAP7D1 MAPRE2 MAPRE3 MARCKS MARCKSL1 MICAL3 MINK1 MPRIP MTM1 MYH10 MYH9 NAV1 NCKAP5L PARD3 PARVA PDLIM2 PDLIM5 PKP2 PLEC PRPF40A RAF1 RCC1 RICTOR SH3D19 SH3KBP1 SHROOM2 SIPA1 SIPA1L1 SLAIN2 SLC16A1 SLC9A3R1 SPAST SPTAN1 SPTBN1 SSH3 TJP1 TLN1 TMSB4X TRIP10 TRPM7 VASP VIM
**Migration and metastasis** IPA: n = 108	ABCC4 ABL1 ADARB1 ADD1 ADRA2A AHNAK AKAP11 AKAP12 ALDOA ANKS1A ANO6 APC ARFGEF2 ARHGAP21 ARHGAP5 ARHGEF7 ASAP1 ATG9A BCR CAP1 CAV1 CCDC88A CD44 CDH11 CHD1 CLASP2 CRTC1 CSNK2B CTNND1 CTTN DAP DAPK3 DEPTOR DPYSL2 DPYSL3 EGLN1 EIF2A ELK3 ENAH EPHA2 FLCN FLNB FNBP1L FNDC3B GIT2 HSP90AA1 ILF3 IRS1 IRS2 KANK1 LIMA1 LMO7 MAGI1 MAP1B MAP4 MAP4K4 MAPKAPK5 MARCKS MARCKSL1 MEIS1 MINK1 MLLT1 MTDH MYH10 MYH9 MYLK MYO9B NAV1 NEXN NFIC PAK2 PARVA PDLIM2 PEAK1 PHLDA2 PKP2 PLA2G4A PLCG1 PLEC PLXNA1 PPFIA1 PRKCD PTK2 PTPN2 PXN RAB27A RAF1 RICTOR ROR2 RPS6 RPS6KA3 RSF1 SH3BP4 SH3KBP1 SIRT1 SLC12A2 SLC16A1 SLC9A3R1 SPAG9 STAP2 SYNJ2 TGFB1I1 TLN1 TMSB4X TNC TNS1 TOP2B TP53BP2

## Abbreviations

cAMP: cyclic AMP
CPM: counts per million
GAP: GTPase activating protein
GEF: guanine nucleotide exchange factor
GO: gene ontology
GPCR: G-protein-coupled receptor
HGSC: high grade serous cancer
H2L: H2L5186303 (LPAR2 antagonist)
LPA: lysophosphatidic acid
LPAR: LPA receptor
MS: mass spectrometry
MYLK: myosin light chain kinase
MYPT1: myosin phosphatase-targeting subunit 1 (PPP1R12A)
OC: ovarian cancer
PKC: protein kinase C
qRT-PCR: quantitative reverse transcription polymerase chain reaction
Ro: Ro6842262 (LPAR1 antagonist)
RNA-Seq: RNA sequencing
siRNA: small interfering RNA
