# Supplementary material for: The lysophosphatidic acid-regulated signal transduction network in ovarian cancer cells and its role in actomyosin dynamics, cell migration and entosis
Source: Theranostics. 2023 Mar 21;13(6):1921–48. doi: 10.7150/thno.81656 (PMC10091871; doi:10.7150/thno.81656)
Supplement: Supplementary file 1 — Supplementary figures and tables, video. [file thnov13p1921s1.zip › Supplemental Figures.pdf]

The lysophosphatidic acid-regulated signal transduction network in ovarian cancer cells and its role in actomyosin dynamics, cell migration and entosis

SUPPLEMENTAL FIGURES

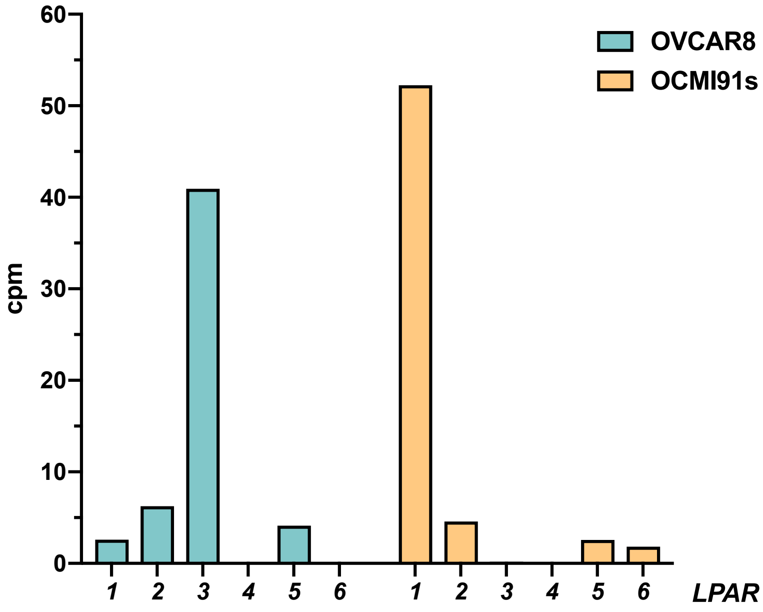

Figure S1. Expression of *LPAR* receptor genes in HGSC cells.

Expression of *LPAR1-6* mRNA in OVCAR8 and OCMI91 cells was analyzed by RNA-Seq. Values represent counts per million (cpm).

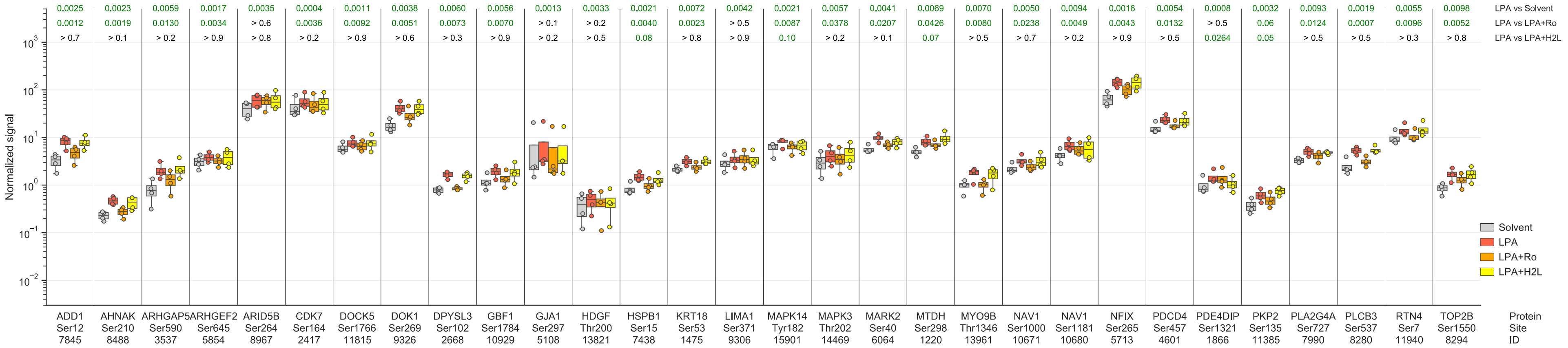

Figure S2. Phosphosites upregulated by LPA in proteins associated with migration and/or metastasis.

The plot includes sites with p <0.01 for LPA versus solvent (as in Fig. 2) in proteins associated with migration and/or metastasis in the Ingenuity Pathway Analysis database. Shown are the median (line), upper and lower quartiles (box), range (whiskers) and values of n=4 replicates (circles). Significance was tested by paired t test: **** p <0.0001; *** p <0.001; ** p <0.01; * p <0.05. Green number indicate p<0.1.

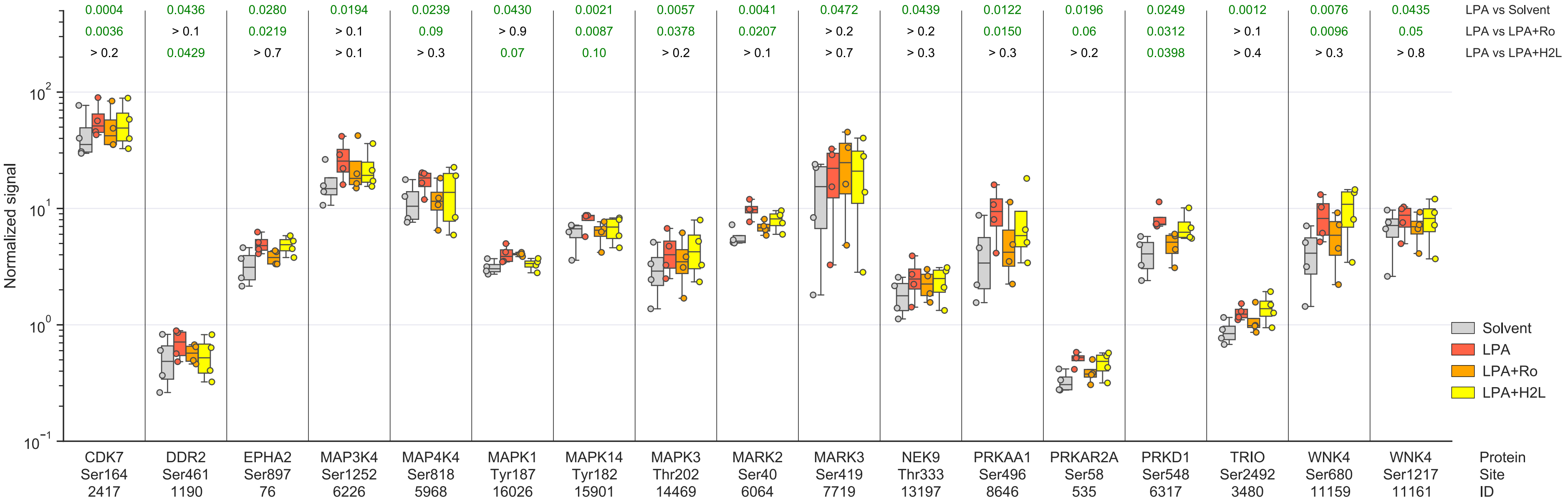

Figure S3. Phosphosites in protein kinases upregulated by LPA

Proteins were identified as described in Fig. 2. The plot includes all sites with $p < 0.05$ for LPA versus solvent. Shown are the median (line), upper and lower quartiles (box), range (whiskers) and values of n=4 replicates (circles). Significance was tested by paired t test: **** $p < 0.0001$; *** $p < 0.001$; ** $p < 0.01$; * $p < 0.05$. Green number indicate $p < 0.1$.

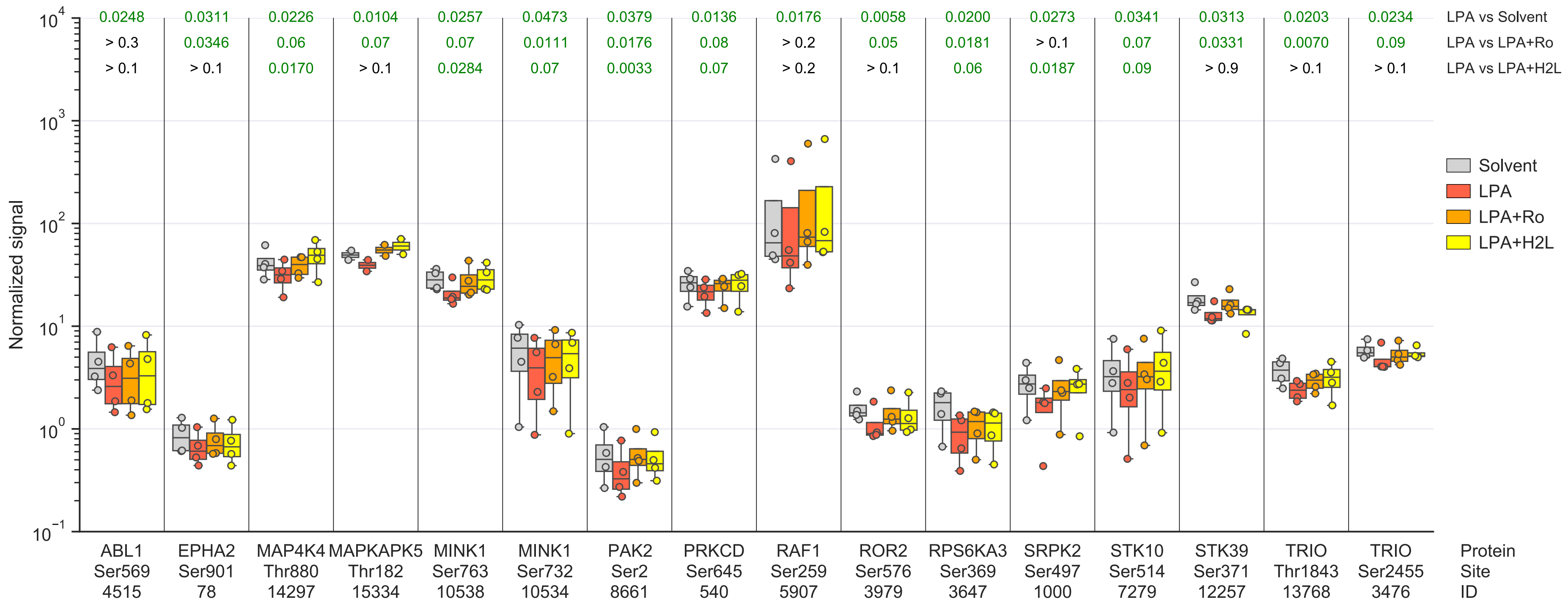

Figure S4. Phosphosites in protein kinases downregulated by LPA.

Proteins were identified as described in Fig. 2. The plot includes all sites with p <0.05 for LPA versus solvent. Shown are the median (line), upper and lower quartiles (box), range (whiskers) and values of n=4 replicates (circles). Significance was tested by paired t test: **** p <0.0001; *** p <0.001; ** p <0.01; * p <0.05. Green number indicate p<0.1.

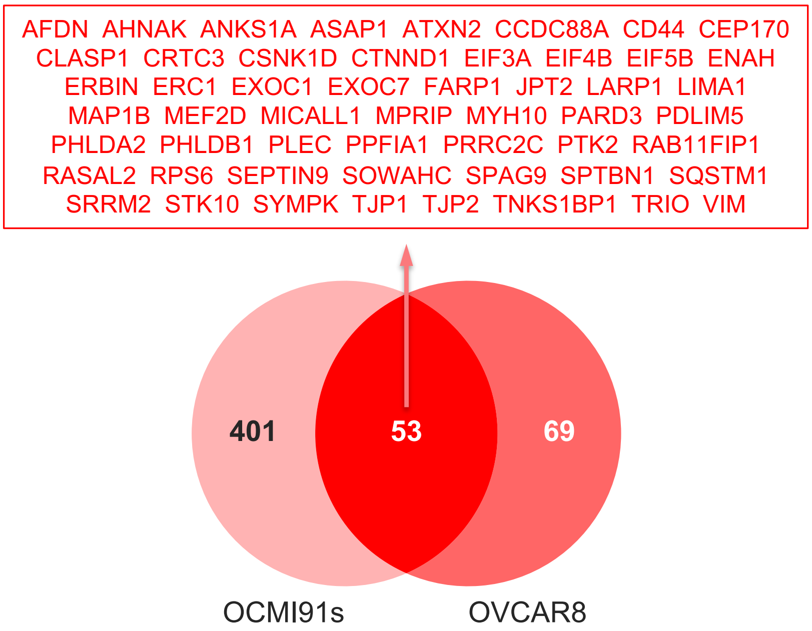

Figure S5. Venn diagrams illustrating the overlap of proteins with LPA-downregulated phosphosites in OCMI91s and OVCAR8 cells.

Threshold: FC<0.83; data in Tables S9 and S15

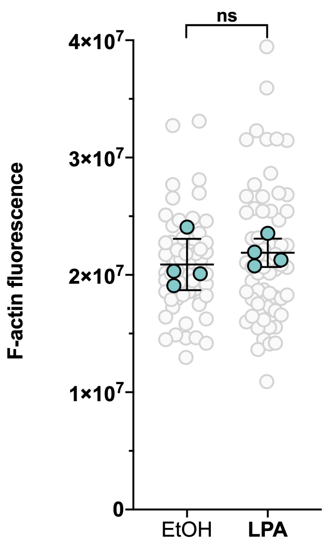

Figure S6. Quantification of F-actin staining in OCMI91s cells treated with LPA or solvent.

Cells were treated with 5 µM LPA or solvent (EtOH) for 1 h and F-actin was visualized by fluorescently labelled phalloidin. The plot shows the mean (green dots) ± SD of n=4 biological replicates with 15 random areas analyzed per replicate (grey dots).

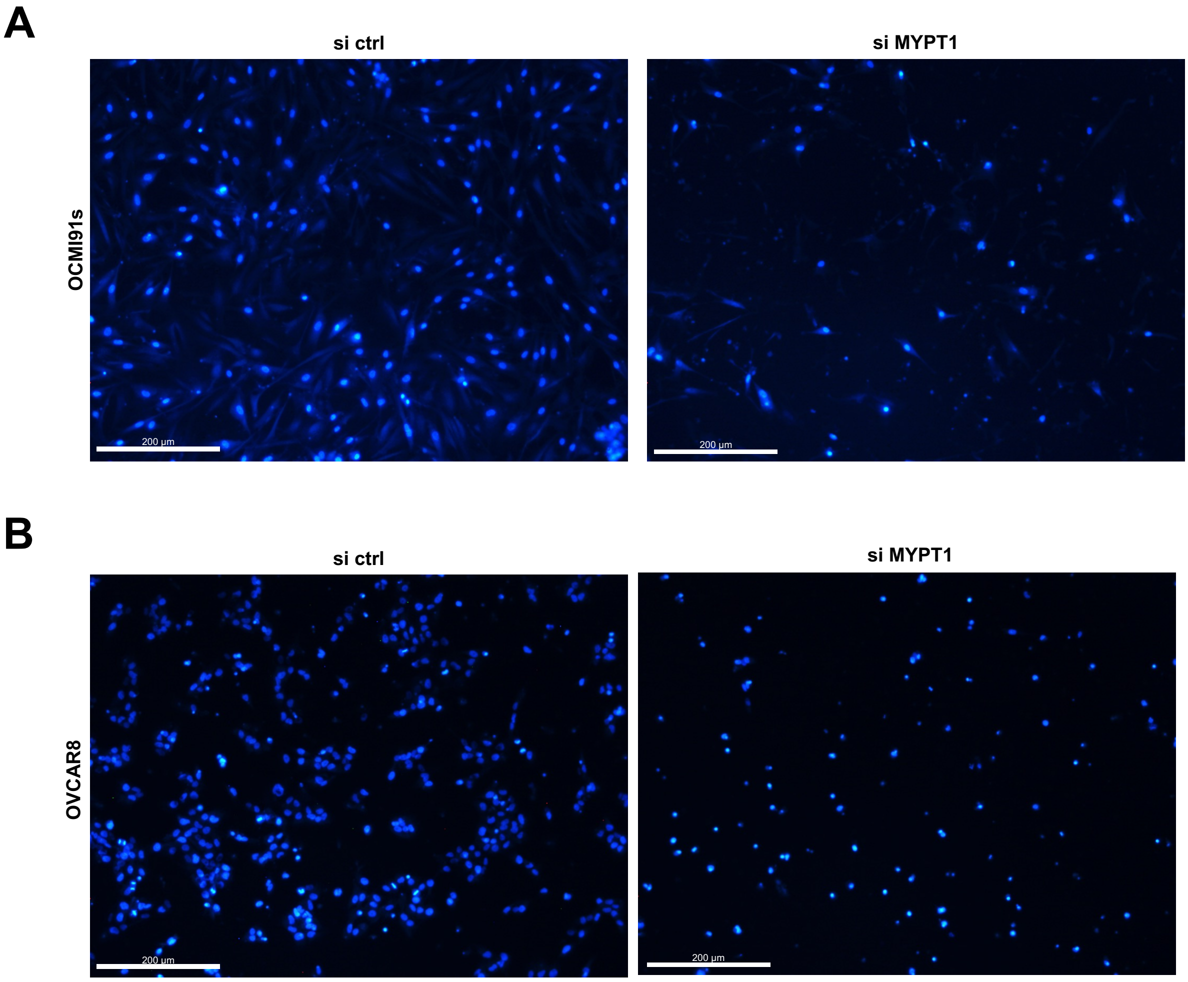

Figure S7. Effect of MYPT1 siRNA on LPA-induced transwell migration.

Representative pictures of transwell assays of OCMI91s cells **(A)** and OVCAR8 cells **(B)** transfected with control siRNA (si ctrl) or siRNA targeting *MYPT1* (si MYPT1) and let migrate towards 5% FCS for 8 h **(A)** or towards 10% FCS for 24 h **(B)**. Quantifications are shown in Fig. 8A and B.

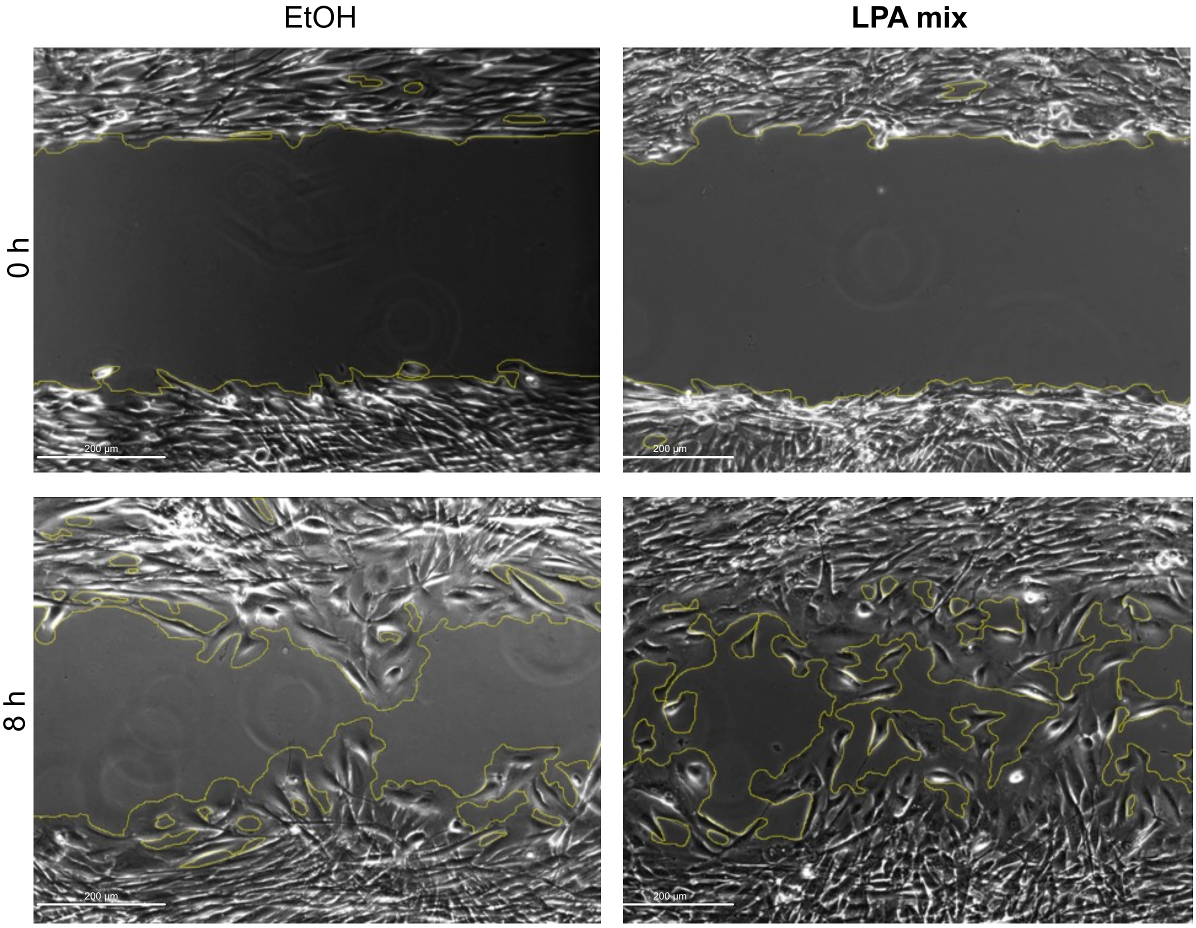

Figure S8. LPA-induced wound healing capacity of OCMI91s cells.

Representative images of wound healing assays. OCMI91s cells were treated with 5 µM LPA mix or solvent (EtOH) for 8 h and the wound closure quantified in 8C. Scale bar: 200 µm.

-

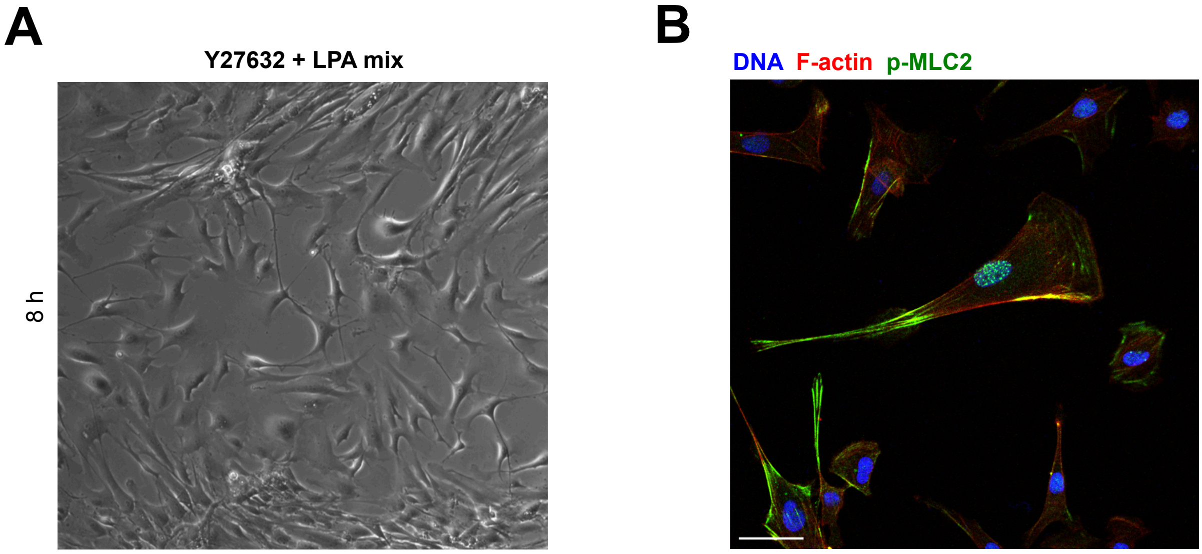

Figure S9. Defective tail retraction of Y27632 pretreated cells

(**A)** OCMI91s cells were treated with 10 μM Y27632 for 30 min before stimulation with 5 μM LPA mix or EtOH for 8 h. Shown is a representative image for the analysis of wound closure after 8 h.

(B) Immunofluorescence staining of migrating OCMI91s cell stimulated with LPA with strong phosphorylation of MLC2 in the tail compartment.

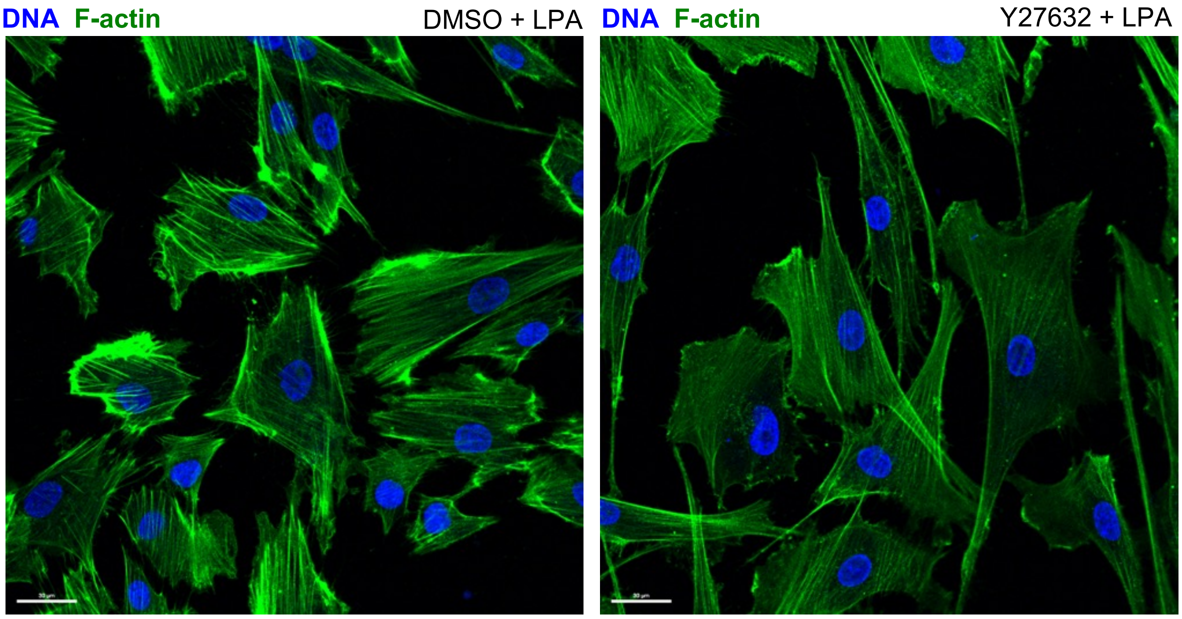

Figure S10. Effects of ROCK inhibition on the actin cytoskeleton and migration of OCMI91s cells.

F-actin was stained with fluorescently labelled phalloidin in cells stimulated with 5 μM LPA for 1 h preincubated for 30 min with DMSO (solvent) or the ROCK inhibitor Y27632. Representative images are shown. Scale bar: 30 μM.

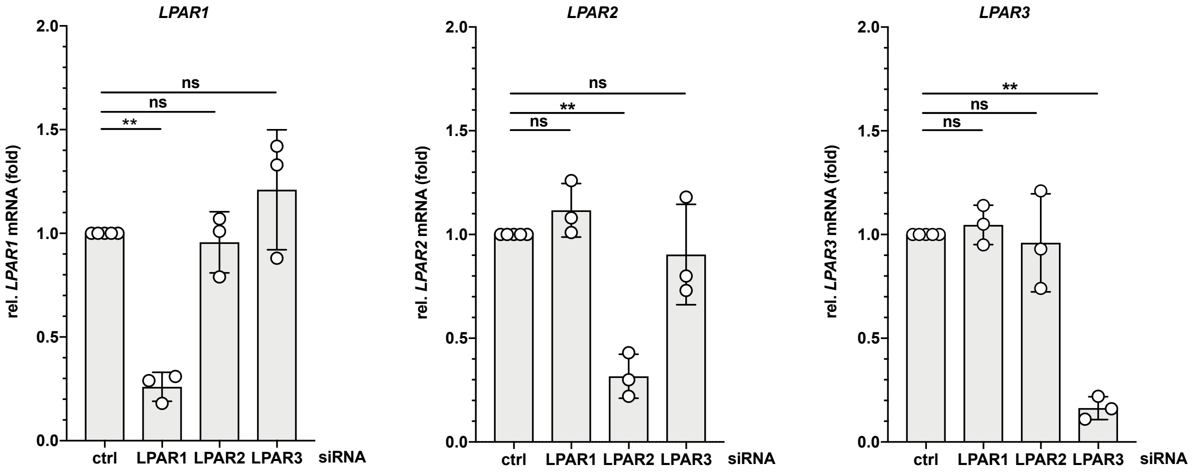

Figure S11. Validation of *LPAR1, LPAR2* and *LPAR3* siRNA.

OVCAR8 cells were double-transfected either with siRNA control (si ctrl) or siRNA targeting *LPAR1, LPAR2 or LPAR3* for 72 h. *LPAR1, LPAR2* and *LPAR3* mRNAs were analyzed by RT-qPCR. Data are presented as fold change to control siRNA (n=3 biological replicates). Shown is the mean ± SD. Asterisks indicate p values determined by two-sided, paired t-test: ** p <0.01; ns: not significant.

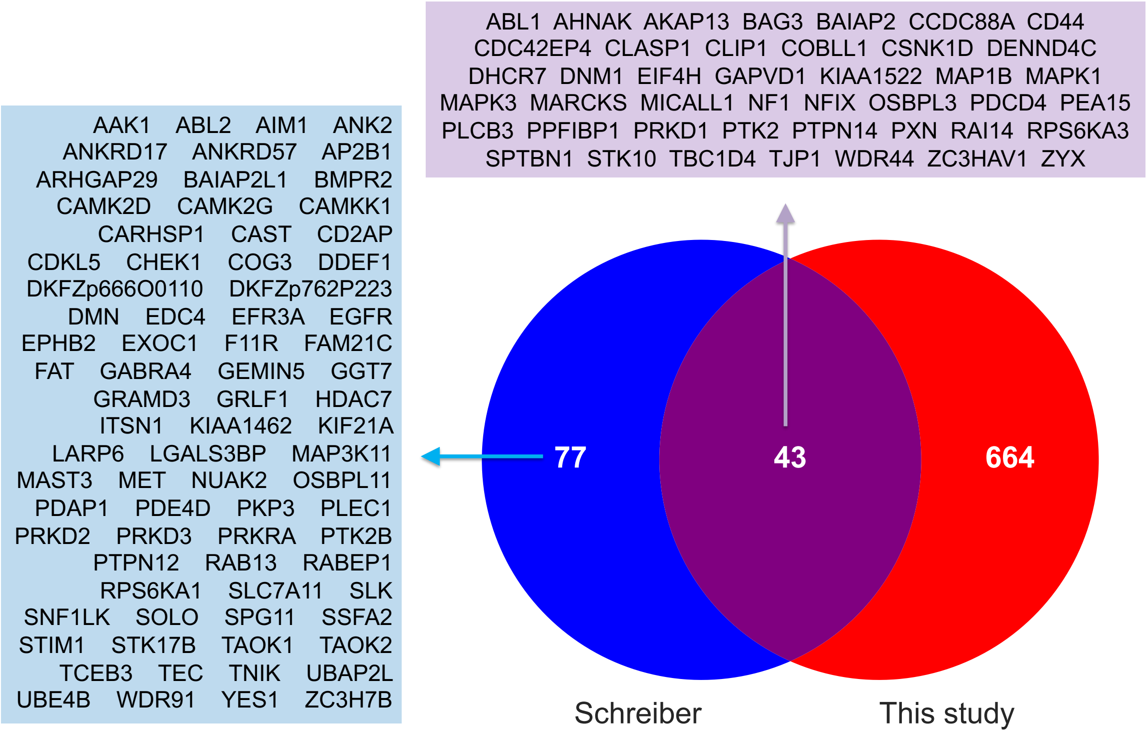

Figure S12. Comparison of LPA-regulated phosphoproteins identified in A498 kidney carcinoma cells (Schreiber et al., 2010) and in patient derived HGSC cells (present study).

The Venn diagram shows the combined up- and downregulated genes.

Reference:

Schreiber, T.B., Mausbacher, N., Keri, G., Cox, J., Daub, H. An integrated phosphoproteomics work flow reveals extensive network regulation in early lysophosphatidic acid signaling. *Mol Cell Proteomics* 2010;**9**:1047-1062.

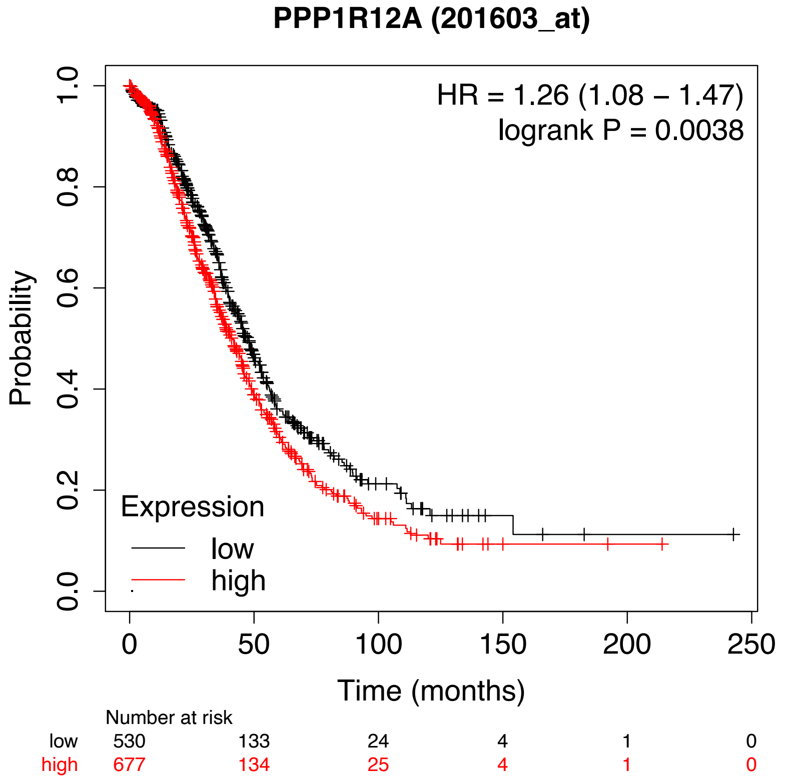

Figure S13. Association of *MYPT1 (PPP1R12A)* expression with overall survival of ovarian serous carcinoma.

The plot was generated by the web-based Kaplan-Meier Plotter (Gyorffy el al., 2012) at http://kmplot.com/analysis.

Settings: best-fit split, JetSet best probe set, serous histology, all stages, all grades.

Reference:

Gyorffy, B., Lanczky, A. & Szallasi, Z. Implementing an online tool for genome-wide validation of survival-associated biomarkers in ovarian-cancer using microarray data from 1287 patients. Endocr Relat Cancer 19, 197–208 (2012).
